# Adaptability and production potential of sorghum in cool regions: heterosis analysis of parameters of light response and CO_2_ response curves

**DOI:** 10.3389/fpls.2026.1905153

**Published:** 2026-08-19

**Authors:** Renjie Zhao, Zexin Qi, Chen Xu, Yu Zhang, Hongyou Zhang, Yang Sun, Jianghong Wang, Zhian Zhang, Ziyang Zhou

**Affiliations:** 1 Institute of Crop Germplasm Resources, Jilin Academy of Agricultural Sciences, Northeast Agricultural Research Center of China, Gongzhuling, China; 2 Agronomy College, Jilin Agricultural University, Jilin, Changchun, China; 3 Institute of Agricultural Resources and Environment, Jilin Academy of Agricultural Sciences, Northeast Agricultural Research Center of China, Changchun, China

**Keywords:** combining ability, heterosis, hybrid F1, photosynthetic response, sorghum, yield

## Abstract

**Introduction:**

Improving photosynthetic performance may contribute to the productivity and adaptation of hybrid sorghum in cool regions. This study aimed to characterize stage-specific photosynthetic performance, heterosis, combining ability, genetic effects, and yield performance in sorghum hybrids and their parental lines.

**Methods:**

Six maternal lines, six restorer lines, and 36 F_1_ hybrids generated using a North Carolina II mating design were evaluated in field experiments conducted in 2021 and 2022. Leaf light- and CO_2_-response curves were measured at the jointing, flowering, and maturity stages. Photosynthetic parameters, heterosis, general combining ability (GCA), specific combining ability (SCA), variance components, heritability, heterotic grouping, and grain yield were analyzed.

**Results:**

Photosynthetic performance of the F_1_ hybrids varied among developmental stages. At the jointing and flowering stages, the hybrids generally exhibited higher light- or CO_2_-saturated photosynthetic capacity, Rubisco carboxylation capacity, and electron-transport capacity than one or both parental groups, together with relatively low respiratory or photorespiratory carbon loss. However, apparent quantum efficiency (AQE) was lower in the hybrids at jointing, and several photosynthetic advantages were not maintained at maturity. Carboxylation efficiency (CE), maximum Rubisco carboxylation rate (Vcmax), and maximum electron-transport rate (Jmax) showed positive average mid-parent and high-parent heterosis, whereas photorespiration rate (Rp) and CO_2_ compensation point (CCP) showed negative average heterosis. TAM428A, I15A, L407A, JiR107, T40, and Ji318R showed complementary GCA advantages, while Ji2055A × T40, TAM428A × T40, TAM428A × 0-30, and I15A × 0-30 exhibited favorable SCA effects for multiple traits. Except for dark respiration rate (Rd), SCA accounted for more than 70% of the total combining-ability variance for all response parameters. Broad-sense heritability exceeded 89%, whereas narrow-sense heritability was generally low, indicating a major contribution of non-additive genetic effects. The mean grain yield of the F_1_ hybrids was 50.54% and 54.10% higher than that of the restorer lines in 2021 and 2022, respectively.

**Discussion:**

The results demonstrate that photosynthetic advantages in hybrid sorghum are strongly dependent on developmental stage and are largely influenced by non-additive genetic effects. Integrating stage-specific photosynthetic evaluation, parental GCA, cross-specific SCA, and multi-environment yield testing may improve the identification and selection of promising sorghum parents and hybrids for cool-region production.

## 1 Introduction

Sorghum [*Sorghum bicolor* (L.) Moench] is a climate-resilient C_4_ cereal used for food, feed, brewing, biomass, and bioenergy production. Its high photosynthetic efficiency, water-use efficiency, and tolerance to drought, salinity, heat, and low-fertility soils make it valuable for agriculture in marginal environments and under increasingly variable climates (Amombo et al., 2022; Kugedera et al., 2022; Baloch et al., 2023). Grain sorghum is an important raw material for the brewing industry in China, whereas sweet sorghum has considerable potential for biomass and renewable-energy production (Jankowski et al., 2020; Zhou et al., 2020). Nevertheless, sorghum originated in warm regions and is more sensitive to chilling than many temperate cereals. Low temperature can restrict germination and establishment, disrupt photosynthetic metabolism, delay development, and impair reproductive growth, thereby limiting the expansion and stable production of sorghum in cool regions (Emendack et al., 2021; Vera Hernández et al., 2023). Substantial genotypic variation in cold tolerance and photosynthetic performance indicates that these limitations can be alleviated through genetic improvement. Identifying genotypes that combine cool-region adaptability with efficient light utilization and CO_2_ assimilation is therefore an important objective of sorghum breeding.

Heterosis has been a major force driving the improvement and commercial production of sorghum. The discovery and utilization of cytoplasmic male sterility established an efficient three-line system involving male-sterile, maintainer, and fertility-restorer lines and enabled large-scale hybrid seed production (Stephens and Holland, 1954). Hybrid sorghum breeding subsequently contributed to improvements in yield, maturity, plant architecture, stress adaptation, and quality. In China, continuing improvement of cytoplasmic male-sterility systems, dwarf germplasm, and parental lines has promoted the development of high-performing hybrids (Meng et al., 2025). Parental value, however, cannot be judged solely from parental performance. General combining ability (GCA) reflects the average contribution of a parent across crosses and is mainly associated with additive effects, whereas specific combining ability (SCA) describes the performance of an individual cross beyond that expected from its parents and is often associated with dominance and epistatic effects (Griffing, 1956). Recent studies have shown that combining heterosis, GCA, SCA, genetic parameters, and multi-environment evaluation improves the identification of superior sorghum parents and crosses (Maulana et al., 2023; Volpato et al., 2024; Zhang et al., 2024).

The physiological basis of yield heterosis is complex because yield is the final result of carbon assimilation, respiratory consumption, assimilate transport, sink formation, and environmental adaptation throughout crop development. Photosynthesis supplies most of the carbon and energy required for biomass accumulation and grain filling, but a high instantaneous net photosynthetic rate does not necessarily result in high yield when respiratory losses, developmental stage, canopy structure, and source–sink coordination are not considered. Consequently, a single gas-exchange measurement under one light intensity and CO_2_ concentration may provide an incomplete description of the photosynthetic advantage of a hybrid. Sorghum possesses extensive natural variation in gas exchange, chlorophyll fluorescence, water-use efficiency, and photosynthetic responses to drought and salinity (Ortiz and Salas-Fernandez, 2022; Amombo et al., 2024; Al-Salman et al., 2024). Sorghum F_1_ hybrids have also shown heterosis for net photosynthetic rate, stomatal conductance, transpiration, carbon-metabolism-related enzyme activities, biomass, and yield components at flowering (Zhao et al., 2024). These findings support the use of photosynthetic traits in hybrid evaluation, while indicating that photosynthetic heterosis should be characterized across developmental stages rather than inferred from a single-point measurement.

Photosynthetic light-response and CO_2_-response curves provide complementary approaches for resolving different components of leaf carbon assimilation. Light-response curves describe changes in net photosynthetic rate from light limitation to saturation and provide parameters related to low-light utilization, respiratory carbon loss, maximum photosynthetic capacity, and light adaptation. CO_2_-response curves characterize the response of photosynthesis to intercellular CO_2_ availability and provide information on carboxylation efficiency, CO_2_ compensation and saturation, photorespiration, Rubisco carboxylation capacity, and electron-transport capacity. Together, these parameters offer a more multidimensional assessment of photosynthetic performance than measurements made under a single environmental condition (Ye, 2010; Ye and Zhao, 2010; Xu et al., 2014; Wang et al., 2023). Recent work in sweet sorghum further showed that short-term photosynthetic and water-use-efficiency responses to light are modified by temperature and CO_2_ concentration, emphasizing the environmental sensitivity of response-curve traits (Yang et al., 2024). Curve-derived parameters may therefore distinguish hybrids with enhanced photochemical efficiency, carboxylation, and electron transport from those whose apparent photosynthetic advantage is accompanied by greater respiratory or photorespiratory carbon loss.

The genetic improvement of photosynthesis is receiving increasing attention because natural variation in photosynthetic traits may provide breeding targets for enhanced productivity and environmental resilience (Khan et al., 2024; Ali et al., 2025). However, these traits are dynamic and strongly affected by genotype, developmental stage, and measurement environment. Their breeding value depends not only on phenotypic differences but also on the relative contributions of additive and non-additive genetic effects and the consistency of trait expression across stages. Existing sorghum studies have clarified the genetic control of several photosynthetic traits under stress and documented photosynthetic heterosis at flowering (Ortiz and Salas-Fernandez, 2022; Zhao et al., 2024). Nevertheless, to our knowledge, limited information is available on how complete sets of light-response and CO_2_-response curve parameters vary among male-sterile lines, restorer lines, and their F_1_ hybrids across successive growth stages. Even less is known about the heterosis, combining ability, heritability, heterotic grouping, and yield associations of these traits when evaluated within the same mating design under cool-region field conditions.

In this study, six male-sterile lines, six restorer lines, and their 36 F_1_ combinations generated using an NCII mating design were evaluated at the jointing, flowering, and maturity stages. We hypothesized that: (1) the F_1_ hybrids would display stage-dependent advantages in light utilization and CO_2_ assimilation, especially at the jointing and flowering stages; (2) favorable heterosis would involve higher photosynthetic capacity, carboxylation efficiency, and electron-transport capacity together with lower respiratory and photorespiratory carbon loss; (3) non-additive genetic effects would contribute substantially to variation in most response-curve parameters; and (4) hybrids with favorable photosynthetic response parameters would tend to exhibit superior yield performance. Accordingly, the objectives were to compare light-response and CO_2_-response curves and their fitted parameters among the hybrids and parents at three growth stages; quantify mid-parent and high-parent heterosis; estimate GCA, SCA, heritability, and related genetic parameters; classify the parents into heterotic groups; and evaluate relationships between response-curve parameters and yield. This study provides a physiological and genetic basis for selecting superior parents and hybrid combinations and for improving high-photosynthetic-efficiency sorghum breeding in cool regions.

## 2 Materials and methods

### 2.1 Test materials

Six cytoplasmic male-sterile (A) lines, Ji2055A, TAM428A, 521A, I15A, 170A, and L407A, and six fertility-restorer (R) lines, Nan133, Ji318R, T40, 0-30, 501R, and JiR107, were used as parental materials. The six male-sterile lines were each crossed with the six restorer lines according to a North Carolina II (NC II) mating design, generating 36 F_1_ hybrid combinations. Thus, a total of 48 genotypes, comprising 12 parental lines and 36 F_1_ hybrids, were included in the experiment. The parental lines and corresponding hybrid combinations are presented in **Table 1**. All plant materials were provided by the Institute of Crop and Resource Research, Jilin Academy of Agricultural Sciences, China.

**Table 1:** Parental lines and 36 F_1_ hybrid combinations generated using the North Carolina II mating design.

Male sterile lines	Restorer lines					
	Nan133	Ji318R	T40	0-30	501R	JiR107
Ji2055A	Ji2055A×Nan133	Ji2055A×Ji318R	Ji2055A×T40	Ji2055A×0-30	Ji2055A×501R	Ji2055A×JiR107
TAM428A	TAM428A×Nan133	TAM428A×Ji318R	TAM428A×T40	TAM428A×0-30	TAM428A×501R	TAM428A×JiR107
521A	521A×Nan133	521A×Ji318R	521A×T40	521A×0-30	521A×501R	521A×JiR107
I15A	I15A×Nan133	I15A×Ji318R	I15A×T40	I15A×0-30	I15A×501R	I15A×JiR107
170A	170A×Nan133	170A×Ji318R	170A×T40	170A×0-30	170A×501R	170A×JiR107
L407A	L407A×Nan133	L407A×Ji318R	L407A×T40	L407A×0-30	L407A×501R	L407A×JiR107

### 2.2 Experimental design

The 36 F_1_ hybrid combinations were produced from October 2020 to April 2021 at the Nanfan Breeding Base of the Jilin Academy of Agricultural Sciences in Foluo Town, Ledong Li Autonomous County, Hainan Province, China (18°35′ N, 108°43′ E). The six male-sterile lines were crossed with the six fertility-restorer lines according to the North Carolina II mating design described above. Field experiments were conducted in 2021 and 2022 at the Sorghum Germplasm Innovation Experimental Base of the Jilin Academy of Agricultural Sciences in Gongzhuling District, Jilin Province, China (43°29′ N, 124°48′ E). A total of 48 genotypes, comprising 36 F_1_ hybrids and 12 parental lines, were evaluated. For the field evaluation, the corresponding maintainer (B) lines were planted in place of the six cytoplasmic male-sterile (A) lines, whereas the six fertility-restorer lines were planted directly as paternal parents. The experiment was arranged in a randomized complete block design with three replicates, resulting in a total of 144 plots. Each plot consisted of six rows, with a row spacing of 0.65 m and a row length of 5 m, giving a plot area of 19.5 m². Seeds were sown on May 13, and seedlings were thinned at the five-leaf stage to a final planting density of 120,000 plants ha^−^¹. Plants were harvested on September 29. All plots received the same conventional field management practices throughout the growing season. Before the field experiment, soil samples were collected from the 0–40 cm soil layer. The soil contained 22.40 g kg^−^¹ organic matter, 75.70 mg kg^−^¹ available nitrogen, 42.10 mg kg^−^¹ available phosphorus, and 120.30 mg kg^−^¹ available potassium, with a pH of 6.43.

### 2.3 Measurement of light- and CO_2_-response curves

Leaf gas-exchange measurements were conducted at the jointing, flowering, and maturity stages using an LI-6800 portable photosynthesis system (LI-COR Biosciences, Lincoln, NE, USA). Measurements were performed on clear, cloudless days between 08:00 and 12:00. For each genotype, one representative plant with uniform growth was selected from each of the three replicate plots, providing three independent biological replicates. The uppermost fully expanded leaf was measured at the jointing stage, whereas the flag leaf was measured at the flowering and maturity stages.

For the light-response measurements, the leaf chamber temperature was maintained at 27°C, the relative humidity was maintained at approximately 65%, and the reference CO_2_ concentration was maintained at 400 μmol mol^−^¹. Net photosynthetic rate (Pn) was measured at 15 photosynthetic photon flux density levels of 0, 30, 70, 100, 150, 200, 300, 500, 700, 900, 1,200, 1,500, 1,800, 2,100, and 2,400 μmol m^−^² s^−^¹. After Pn had stabilized at each light level, the corresponding gas-exchange data were recorded. The measured data were fitted using the modified rectangular hyperbola model described by Ye and Zhao (2010). Apparent quantum efficiency (AQE), dark respiration rate (Rd), light compensation point (LCP), light saturation point (LSP), and maximum net photosynthetic rate under light-saturated conditions (Amax) were estimated from the fitted light-response curves.

For the CO_2_-response measurements, the photosynthetic photon flux density was maintained at the saturating light level determined from the light-response measurements conducted on the same measurement day. The leaf chamber temperature and relative humidity were maintained at 27°C and approximately 65%, respectively. The reference CO_2_ concentration in the leaf chamber was adjusted sequentially to 400, 300, 200, 100, 0, 400, 600, 800, 1,000, 1,200, 1,500, and 1,800 μmol mol^−^¹. After Pn had stabilized at each reference CO_2_ concentration, Pn and the corresponding intercellular CO_2_ concentration (Ci) were recorded. The second measurement at 400 μmol mol^−^¹ was included to allow the leaf to recover from exposure to low CO_2_ before the reference CO_2_ concentration was progressively increased.

The relationship between Pn and Ci was fitted using the modified rectangular hyperbola model described by Ye (2010). Carboxylation efficiency (CE), photorespiration rate (Rp), CO_2_ compensation point (CCP), and maximum net photosynthetic rate under CO_2_-saturated conditions (CSPn) were estimated from the fitted CO_2_-response curves. The maximum rate of Rubisco carboxylation (Vcmax) and maximum electron transport rate (Jmax) were also calculated. For each genotype and growth stage, measurements obtained from the three replicate plots were treated as independent biological replicates and were used for subsequent statistical analyses.

### 2.4 Classification of heterotic groups

For the phenotypic clustering analysis, the mean values of all measured traits for each parental line were standardized, and the Euclidean distances among the parental lines were calculated using the following formula:


$${\text{ED}}_{\text{jk}}=\sqrt{\sum_{\text{i}=1}^{\text{n}}{\left({\text{x}}_{\text{ij}}-{\text{x}}_{\text{ik}}\right)}^{2}}$$

For the GCA-based clustering analysis, the general combining ability effects of each trait were used to calculate the Euclidean distances among the parental lines. The calculation method was the same as that used for the phenotypic clustering analysis.

### 2.5 Estimation of heterosis, combining ability, and genetic parameters

Mid-parent heterosis (MPH) and high-parent heterosis (HPH) were calculated for each light- and CO_2_-response curve parameter using the mean phenotypic values of the F_1_ hybrids and their corresponding parents. The calculations were performed as follows:


$$\text{MPH }(\%)=[({\text{F}}_{1}-\text{MP})/\text{MP}]\times 100$$

$$\text{HPH }(\%)=[({\text{F}}_{1}-\text{HP})/\text{HP}]\times 100$$

where F_1_ represents the mean phenotypic value of an F_1_ hybrid, MP represents the mean phenotypic value of its two parents, and HP represents the higher phenotypic value of the two parents. Positive heterosis values indicate that the F_1_ hybrid had a higher value than the corresponding parental reference, whereas negative values indicate that the hybrid had a lower value. The biological significance of positive and negative heterosis was interpreted according to the characteristics of each trait because lower values of Rd, Rp, LCP, and CCP may indicate reduced respiratory or photorespiratory carbon loss.

General combining ability (GCA) and specific combining ability (SCA) were estimated according to the North Carolina II mating design. The GCA effects of the female and male parents and the SCA effect of each hybrid combination were calculated as follows:


$${\text{g}}_{\text{i}}={\bar{\text{X}}}_{\text{i}}-\bar{\text{X}}$$

$${\text{g}}_{\text{j}}={\bar{\text{X}}}_{\text{j}}-\bar{\text{X}}$$

$${\text{s}}_{\text{ij}}={\bar{\text{X}}}_{\text{ij}}-{\bar{\text{X}}}_{\text{i}}-{\bar{\text{X}}}_{\text{j}}+\bar{\text{X}}$$

where g_i_ and g_j_ represent the GCA effects of the ith female parent and the jth male parent, respectively; s_ij_ represents the SCA effect of the hybrid derived from the ith female parent and the jth male parent; ${\bar{\text{X}}}_{\text{ij}}$ represents the mean phenotypic value of the corresponding hybrid; ${\bar{\text{X}}}_{\text{i}}$ and ${\bar{\text{X}}}_{\text{j}}$ represent the mean phenotypic values of all hybrids involving the ith female parent and the jth male parent, respectively; and $\bar{\text{X}}$ represents the overall mean phenotypic value of the 36 F_1_ hybrids.

Analysis of variance based on the North Carolina II mating design was conducted to estimate the GCA variance components of the female and male parents, the SCA variance component associated with the female × male interaction, and the environmental variance component. The proportional contributions of GCA and SCA to the genetic variation in each light- and CO_2_-response curve parameter were subsequently calculated. Broad-sense heritability (H²B) and narrow-sense heritability (H²N) were estimated from the corresponding variance components. Combining ability effects, variance components, and heritability were analyzed using DPS version 7.05.

### 2.6 Yield measurement

At physiological maturity, 10 representative plants were randomly sampled from the central rows of each replicate plot, with border plants excluded to minimize edge effects. Thus, 30 plants were evaluated for each genotype across the three replicate plots. The sampled panicles were harvested, air-dried, and manually threshed, after which the grain weight per panicle was determined. Grain yield was estimated from the mean grain weight per panicle and the final plant density and was converted to yield per unit area. The same sampling and measurement procedures were used in both experimental years.

### 2.7 Curve fitting and statistical analysis

The light-response and CO_2_-response curves were fitted using the modified rectangular hyperbola model described by Ye and Zhao (2010) and Ye (2010), respectively. The light-response model was expressed as follows:


Pn(I)=α×[(1−βI)/(1+γI)]×I−Rd

where Pn(I) is the net photosynthetic rate at a given photosynthetic photon flux density; I is the photosynthetic photon flux density (μmol m^−^² s^−^¹); α is the initial slope of the light-response curve and represents the apparent quantum efficiency (AQE); β and γ are coefficients of the modified rectangular hyperbola model; and Rd is the dark respiration rate (μmol m^−^² s^−^¹). The fitted curve was used to estimate AQE, Rd, the light compensation point (LCP), the light saturation point (LSP), and the maximum net photosynthetic rate under light-saturated conditions (Amax).

The CO_2_-response model was expressed as follows:


Pn(Ci)=α×[(1−βCi)/(1+γCi)]×Ci−Rp

where Pn(Ci) is the net photosynthetic rate at a given intercellular CO_2_ concentration; Ci is the intercellular CO_2_ concentration (μmol mol^−^¹); α is the initial slope of the CO_2_-response curve and represents the carboxylation efficiency (CE); β and γ are coefficients of the modified rectangular hyperbola model; and Rp is the photorespiration rate (μmol m^−^² s^−^¹). The fitted curve was used to estimate CE, Rp, the CO_2_ compensation point (CCP), and the maximum net photosynthetic rate under CO_2_-saturated conditions (CSPn). The maximum rate of Rubisco carboxylation (Vcmax) and the maximum electron transport rate (Jmax) were calculated from the fitted CO_2_-response data.

Data from the three replicate plots were treated as independent biological replicates. Statistical analyses were performed separately for each growth stage. Differences among genotypes were evaluated using one-way analysis of variance, followed by Duncan’s multiple range test when the analysis of variance indicated a significant effect. Differences were considered significant at P < 0.05 and highly significant at P < 0.01.

Analysis of variance for the North Carolina II mating design and the estimation of combining ability effects, variance components, and heritability were performed using DPS version 7.05. Correlations between photosynthetic response parameters and yield were analyzed using Pearson’s correlation coefficients in SPSS version 22.0. Data were organized using Microsoft Excel 2016, and figures were prepared using Origin 2021.

## 3 Results and analysis

### 3.1 Comparison of leaf light-response curves among F_1_ hybrids and their parents

**Figure 1:**
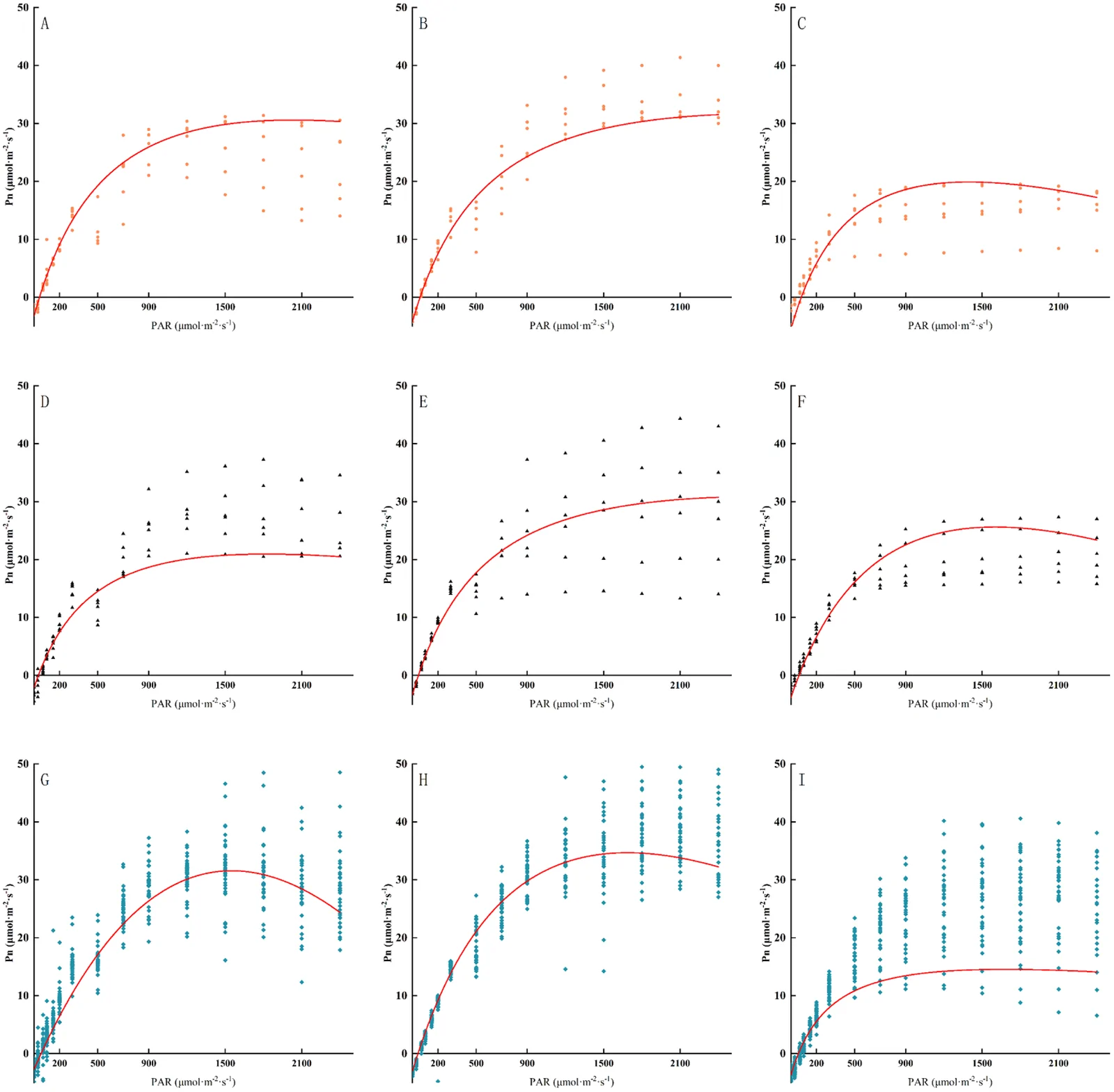
Leaf light-response curves of the maternal parents, paternal parents, and F_1_ hybrids at different growth stages. Panels **(A–C)** show the light-response curves of the maternal parents at the jointing, flowering, and maturity stages, respectively; panels **(D–F)** show those of the paternal parents at the corresponding stages; and panels **(G–I)** show those of the F_1_ hybrids at the corresponding stages.

**Figure 1** showed the responses of net photosynthetic rate (Pn) to increasing photosynthetically active radiation (PAR) in the maternal parents, paternal parents, and F_1_ hybrids at the jointing, flowering, and maturity stages. At low PAR levels (≤200 μmol m^−^² s^−^¹), Pn increased rapidly and approximately linearly with increasing PAR in all three groups. At the jointing stage, the increase in Pn was greatest in the maternal parents, followed by the paternal parents and F_1_ hybrids. At the flowering stage, the F_1_ hybrids showed the most rapid increase in Pn, whereas at the maturity stage, the paternal parents showed a stronger response than the maternal parents and F_1_ hybrids.

As PAR increased from 500 to 1,500 μmol m^−^² s^−^¹, Pn continued to increase and gradually approached light saturation. Within this PAR range, the mean Pn of the F_1_ hybrids gradually exceeded that of the paternal parents, whereas the light-response curves of the parental groups changed more slowly. When PAR exceeded 1,500 μmol m^−^² s^−^¹, Pn tended to stabilize or declined slightly in some parental lines and F_1_ hybrids, suggesting that photosynthesis had reached or exceeded light saturation under high irradiance.

The light-response curves also varied among growth stages. The mean Pn values of the F_1_ hybrids and maternal parents were generally higher at the flowering stage than at the jointing and maturity stages. The F_1_ hybrids showed a particularly strong light response at flowering, whereas their Pn declined at maturity. These results indicated that the light-response characteristics of the F_1_ hybrids varied with developmental stage and that stronger photosynthetic performance was observed during flowering.

### 3.2 Comparison of fitted light-response parameters among F_1_ hybrids and their parents

**Table 2** showed the fitted light-response parameters of the maternal parents, paternal parents, and F_1_ hybrids at the jointing, flowering, and maturity stages. Statistical comparisons were performed among the three genotype groups within each growth stage.

**Table 2:** Fitted light-response parameters of the maternal parents, paternal parents, and F_1_ hybrids at different growth stages.

Stage	Genotypes	AQE (mol mol^−^¹)	Rd	Amax	LSP	LCP
			(μmol·m^-2^·s^-1^)	(μmol·m^-2^·s^-1^)	(μmol·m^-2^·s^-1^)	(μmol·m^-2^·s^-1^)
Jointing stage	Maternal parent	0.041a	3.53a	28.79a	2121.12a	30.78a
	Paternal parent	0.039a	3.66a	20.25b	1472.24b	28.36a
	F_1_ hybrids	0.031b	2.57b	29.24a	1500.54b	33.74a
Flowering stage	Maternal parent	0.061ab	3.28a	31.43b	2378.54a	49.70a
	Paternal parent	0.057b	3.23a	30.25b	2022.00 b	49.08a
	F_1_ hybrids	0.066a	2.94a	37.60a	1892.24b	40.62b
Maturity stage	Maternal parent	0.058a	2.98a	19.58b	1455.71b	41.33a
	Paternal parent	0.055a	3.31a	24.36a	1687.84a	38.48a
	F_1_ hybrids	0.057a	2.24b	14.49c	1272.55c	40.42a

Values followed by different lowercase letters within the same growth stage and parameter are significantly different at P < 0.05.

At the jointing stage, the apparent quantum efficiency (AQE) of the F_1_ hybrids was significantly lower than those of both parental groups, whereas the maternal and paternal parents did not differ significantly from each other. The dark respiration rate (Rd) of the F_1_ hybrids was also significantly lower than those of both parents. The maximum net photosynthetic rate under light-saturated conditions (Amax) of the F_1_ hybrids did not differ significantly from that of the maternal parents but was significantly higher than that of the paternal parents. The light saturation point (LSP) of the maternal parents was significantly higher than those of the paternal parents and F_1_ hybrids, whereas no significant difference was observed between the paternal parents and F_1_ hybrids. No significant difference in the light compensation point (LCP) was detected among the three genotype groups.

At the flowering stage, the F_1_ hybrids had significantly higher AQE than the paternal parents, whereas their AQE did not differ significantly from that of the maternal parents. No significant difference in Rd was observed among the three genotype groups. The Amax of the F_1_ hybrids was significantly higher than those of both parental groups. The maternal parents had a significantly higher LSP than the paternal parents and F_1_ hybrids, whereas the latter two groups did not differ significantly from each other. In contrast, the LCP of the F_1_ hybrids was significantly lower than those of both parental groups.

At the maturity stage, no significant difference in AQE was observed among the maternal parents, paternal parents, and F_1_ hybrids. The F_1_ hybrids had significantly lower Rd than both parental groups, with reductions of 24.83% and 32.33% relative to the maternal and paternal parents, respectively. The paternal parents had the highest Amax, followed by the maternal parents and F_1_ hybrids, and the differences among the three groups were significant. A similar pattern was observed for LSP, which was significantly highest in the paternal parents, intermediate in the maternal parents, and lowest in the F_1_ hybrids. No significant difference in LCP was detected among the three genotype groups.

Overall, the F_1_ hybrids exhibited lower respiratory carbon loss at all three growth stages, as indicated by their relatively low Rd values. Their light-saturated photosynthetic capacity was comparable to or greater than that of the parents at the jointing and flowering stages but was lower than that of both parental groups at maturity. These results indicated that the light-response characteristics of the F_1_ hybrids varied with developmental stage and that their photosynthetic advantage was mainly observed during the jointing and flowering stages.

### 3.3 Comparison of leaf CO_2_-response curves among F_1_ hybrids and their parents

**Figure 2:**
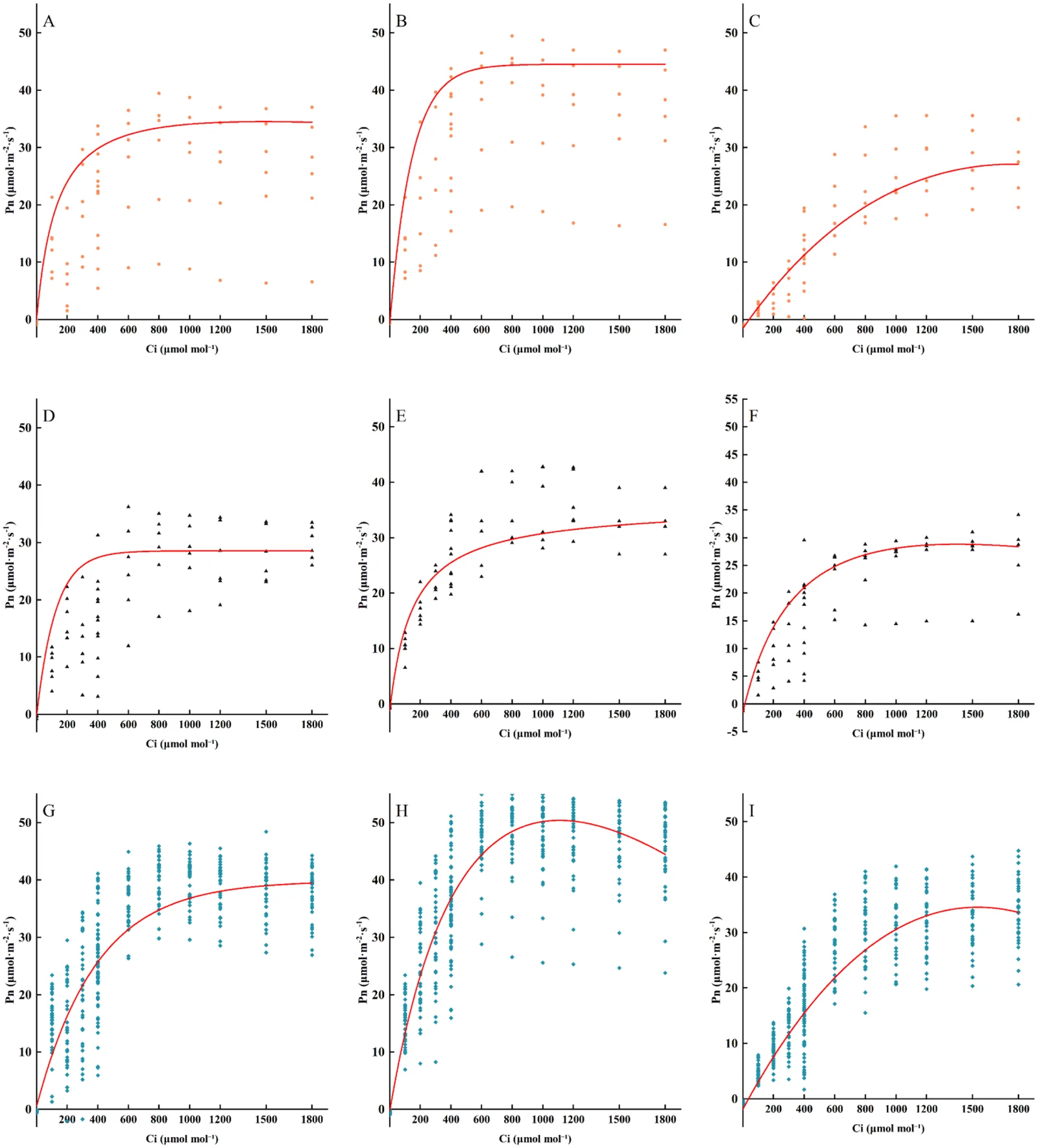
Leaf CO_2_-response curves of the maternal parents, paternal parents, and F_1_ hybrids at different growth stages. Panels **(A–C)** show the CO_2_-response curves of the maternal parents at the jointing, flowering, and maturity stages, respectively; panels **(D–F)** show those of the paternal parents at the corresponding stages; and panels **(G–I)** show those of the F_1_ hybrids at the corresponding stages.

**Figure 2** showed the responses of net photosynthetic rate (Pn) to increasing intercellular CO_2_ concentration (Ci) in the maternal parents, paternal parents, and F_1_ hybrids at the jointing, flowering, and maturity stages. At relatively low Ci levels, Pn increased rapidly with increasing Ci in all three genotype groups. As Ci continued to increase, the rate of increase in Pn gradually declined, and the curves approached CO_2_ saturation.

At the jointing stage, within the Ci range of approximately 0–200 μmol mol^−^¹, the initial increase in Pn was greatest in the maternal parents, followed by the paternal parents and F_1_ hybrids. When Ci exceeded approximately 400 μmol mol^−^¹, the Pn values of the parental groups gradually approached a plateau, whereas those of the F_1_ hybrids continued to increase and reached a relatively high level at approximately 800 μmol mol^−^¹.

At the flowering stage, the initial increase in Pn under low Ci followed the order maternal parents > F_1_ hybrids > paternal parents. As Ci increased, the Pn of the F_1_ hybrids became higher than those of both parental groups over most of the intermediate and high Ci range and reached its highest level at approximately 1,000 μmol mol^−^¹. The F_1_ hybrids therefore showed a stronger CO_2_ response at the flowering stage than the two parental groups.

At the maturity stage, the initial increase in Pn under low Ci was greatest in the paternal parents, followed by the maternal parents and F_1_ hybrids. The paternal parents also maintained relatively high Pn values over the intermediate and high Ci range, whereas the response of the F_1_ hybrids was comparatively weaker than that observed at the jointing and flowering stages.

The CO_2_-response curves varied descriptively among growth stages. The Pn values of the F_1_ hybrids and maternal parents were generally higher at flowering than at jointing and maturity, whereas the paternal parents maintained comparatively strong CO_2_-response capacity at maturity. These results indicated that the CO_2_-assimilation characteristics of the F_1_ hybrids varied with developmental stage and that their strongest response to increasing Ci was mainly observed at flowering.

### 3.4 Comparison of fitted CO_2_-response parameters among F_1_ hybrids and their parents

**Table 3** showed the fitted CO_2_-response parameters of the maternal parents, paternal parents, and F_1_ hybrids at the jointing, flowering, and maturity stages. Statistical comparisons were performed among the three genotype groups within each growth stage.

**Table 3:** Fitted CO_2_-response parameters of the maternal parents, paternal parents, and F_1_ hybrids at different growth stages.

Stage	Genotypes	CE (mol m^−^² s^−^¹)	CSPn	Rp	CCP	Vcmax	Jmax
			(μmol·m^-2^·s^-1^)	(μmol·m^-2^·s^-1^)	(μmol mol^−^¹)	(μmol·m^-2^·s^-1^)	(μmol·m^-2^·s^-1^)
Jointing stage	Maternal parent	0.158a	31.08b	0.378a	6.18b	75.72b	134.25b
	Paternal parent	0.137ab	28.71b	0.329ab	8.71a	62.69c	127.32c
	Hybrid F_1_	0.119b	39.67a	0.285b	4.62c	87.37a	156.75a
Flowering stage	Maternal parent	0.188a	41.52b	0.912a	5.08a	93.79b	156.96b
	Paternal parent	0.142b	35.18c	0.543b	4.67a	73.76c	130.01c
	Hybrid F_1_	0.207a	49.52a	0.495b	2.74b	113.41a	203.05a
Flowering stage	Maternal parent	0.078b	25.49a	0.837a	18.34a	78.95b	150.75a
	Paternal parent	0.110a	28.75a	0.692a	10.04b	82.34a	159.21a
	Hybrid F_1_	0.052b	31.81a	0.774a	17.58a	66.26c	138.94b

Values followed by different lowercase letters within the same growth stage and parameter are significantly different at P < 0.05.

At the jointing stage, the carboxylation efficiency (CE) of the F_1_ hybrids was significantly lower than that of the maternal parents but did not differ significantly from that of the paternal parents. The maximum net photosynthetic rate under CO_2_-saturated conditions (CSPn) of the F_1_ hybrids was significantly higher than those of the maternal and paternal parents by 27.64% and 38.17%, respectively. The photorespiration rate (Rp) of the F_1_ hybrids was 24.60% lower than that of the maternal parents and 13.37% lower than that of the paternal parents; however, the difference was significant only between the F_1_ hybrids and maternal parents. The CO_2_ compensation point (CCP) of the F_1_ hybrids was significantly lower than those of the maternal and paternal parents by 25.24% and 46.96%, respectively. The maximum rate of Rubisco carboxylation (Vcmax) of the F_1_ hybrids was significantly higher than those of the maternal and paternal parents by 15.39% and 39.37%, respectively. Similarly, the maximum electron transport rate (Jmax) of the F_1_ hybrids was significantly higher than those of the maternal and paternal parents by 16.76% and 23.11%, respectively.

At the flowering stage, the CE of the F_1_ hybrids was 10.11% higher than that of the maternal parents, although the difference was not significant, and was significantly higher than that of the paternal parents by 45.77%. The CSPn of the F_1_ hybrids was significantly higher than those of the maternal and paternal parents by 19.27% and 40.76%, respectively. The Rp of the F_1_ hybrids was 45.72% lower than that of the maternal parents and 8.84% lower than that of the paternal parents; however, the difference was significant only between the F_1_ hybrids and maternal parents. The CCP of the F_1_ hybrids was significantly lower than those of the maternal and paternal parents by 46.06% and 41.33%, respectively. The Vcmax of the F_1_ hybrids was significantly higher than those of the maternal and paternal parents by 20.92% and 53.76%, respectively, whereas Jmax was significantly higher by 29.36% and 56.18%, respectively.

At the maturity stage, no significant differences in CSPn or Rp were detected among the maternal parents, paternal parents, and F_1_ hybrids. The CE of the F_1_ hybrids did not differ significantly from that of the maternal parents but was significantly lower than that of the paternal parents. The CCP of the F_1_ hybrids did not differ significantly from that of the maternal parents but was significantly higher than that of the paternal parents. The paternal parents had the highest Vcmax, followed by the maternal parents and F_1_ hybrids, and the differences among the three groups were significant. The Jmax of the F_1_ hybrids was significantly lower than those of both parental groups, whereas no significant difference was observed between the maternal and paternal parents.

### 3.5 Variation in light- and CO_2_-response parameters among parental lines and F_1_ hybrids

**Supplementary Table 1** showed substantial variation in the nine light- and CO_2_-response parameters among the 12 parental lines and 36 F_1_ hybrids. The effects of genotype were highly significant for AQE, Rd, LSP, LCP, CE, CCP, and Jmax (P < 0.01) and significant for Rp and Vcmax (P < 0.05). The coefficients of variation ranged from 9.04% to 28.49%. CE exhibited the greatest variation, with a coefficient of variation of 28.49%, followed by Rd and Rp, with coefficients of variation of 21.86% and 21.69%, respectively. CCP exhibited the lowest coefficient of variation at 9.04%, followed by Jmax at 9.46%.

For the light-response parameters, the mean AQE values of the maternal lines, restorer lines, and F_1_ hybrids were 0.0662, 0.0657, and 0.0627, respectively. The highest AQE values within the three groups were recorded for L407A, JiR107, and I15A × Ji318R, whereas the lowest values were recorded for I15A, 501R, and Ji2055A × 0-30, respectively. The corresponding mean Rd values were 2.81, 3.23, and 3.28 μmol m^−^² s^−^¹. Ji2055A, Ji318R, and 521A × 0–30 had the highest Rd values within their respective groups, whereas I15A, 0-30, and I15A × Ji318R had the lowest values. The mean LSP values of the maternal lines, restorer lines, and F_1_ hybrids were 1,992.24, 2,322.54, and 2,022.00 μmol m^−^² s^−^¹, respectively. Ji2055A, JiR107, and I15A × 0–30 had the highest LSP values, whereas 521A, 501R, and 521A × 0–30 had the lowest values within the three groups. The mean LCP values were 49.69, 40.62, and 49.08 μmol m^−^² s^−^¹ for the maternal lines, restorer lines, and F_1_ hybrids, respectively. The highest LCP values were observed for 170A, Ji318R, and Ji2055A × 0-30, whereas the lowest values were observed for TAM428A, 501R, and I15A × Ji318R, respectively.

For the CO_2_-response parameters, the mean CE values of the maternal lines, restorer lines, and F_1_ hybrids were 0.188, 0.122, and 0.207, respectively. Ji2055A, Nan133, and TAM428A × T40 had the highest CE values within their respective groups, whereas I15A, Ji318R, and Ji2055A × 0–30 had the lowest values. The corresponding mean Rp values were 0.912, 0.543, and 0.495 μmol m^−^² s^−^¹. TAM428A, 501R, and Ji2055A × 501R had the highest Rp values, whereas 170A, 0-30, and Ji2055A × T40 had the lowest values within the three groups. The mean CCP values of the maternal lines, restorer lines, and F_1_ hybrids were 5.089, 4.675, and 2.747 μmol mol^−^¹, respectively. The highest CCP values were recorded for L407A, 501R, and Ji2055A × 0-30, whereas the lowest values were recorded for Ji2055A, 0-30, and 521A × JiR107, respectively.

The mean Vcmax values of the maternal lines, restorer lines, and F_1_ hybrids were 93.79, 73.76, and 113.41 μmol m^−^² s^−^¹, respectively. Ji2055A, T40, and TAM428A × T40 had the highest Vcmax values within the three groups, whereas 170A, Ji318R, and 170A × 0–30 had the lowest values. The corresponding mean Jmax values were 156.96, 130.01, and 203.05 μmol m^−^² s^−^¹. The highest Jmax values were observed for 521A, JiR107, and 170A × Ji318R, whereas the lowest values were observed for 170A, Ji318R, and 170A × T40, respectively.

Overall, the F_1_ hybrids exhibited higher group means for CE, Vcmax, and Jmax and lower group means for Rp and CCP than both parental groups. However, hybrid combinations derived from parents with the highest or lowest values did not consistently exhibit corresponding extreme values. These results indicated substantial variation among the hybrid combinations and suggested that the inheritance of the response-curve parameters could not be explained solely by the phenotypic performance of their parents. Formal estimates of mid-parent and high-parent heterosis are presented in Section 3.6.

### 3.6 Heterosis of light- and CO_2_-response parameters in F_1_ hybrids

**Figure 3:**
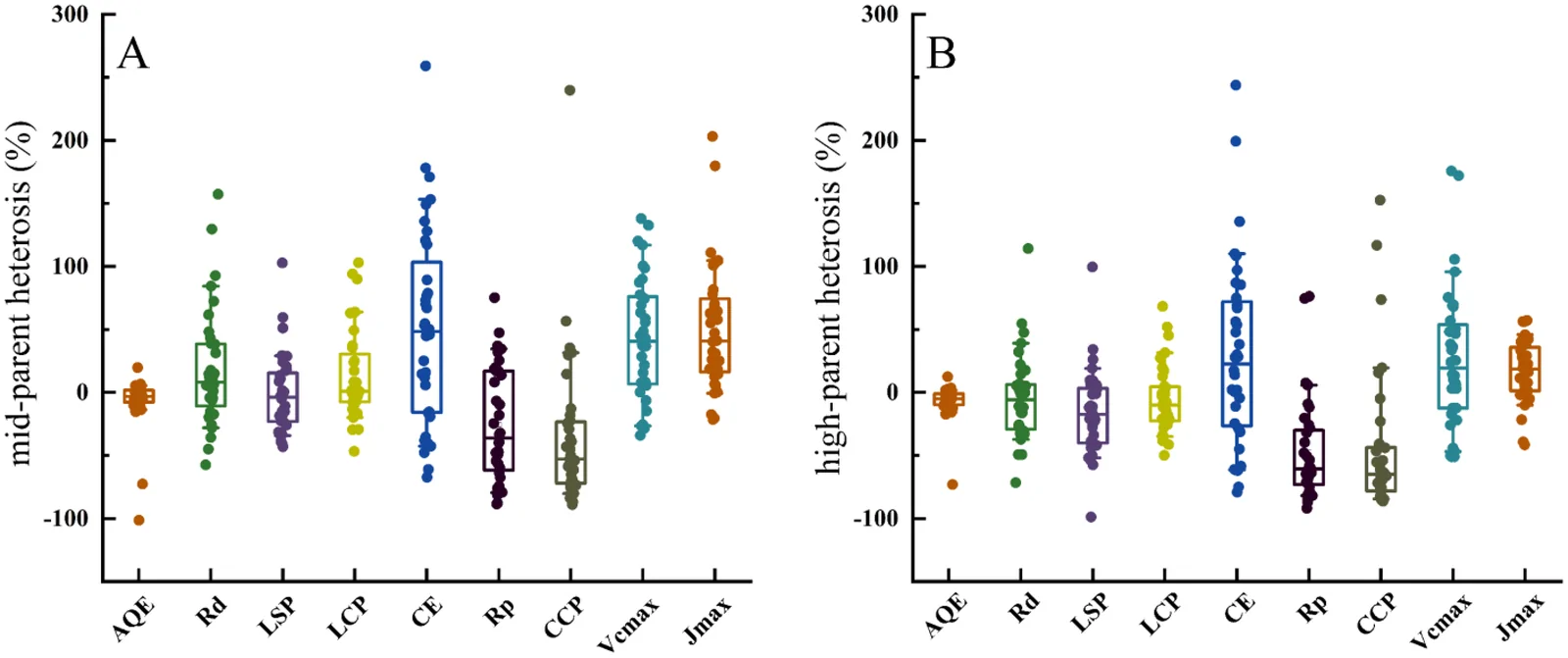
Mid-parent and high-parent heterosis of light- and CO_2_-response parameters in the 36 F_1_ hybrids. **(A)** Mid-parent heterosis of the nine response-curve parameters. **(B)** High-parent heterosis of the nine response-curve parameters.

The mid-parent heterosis of the nine light- and CO_2_-response parameters in the 36 F_1_ hybrids is shown in **Figure 3A**. The magnitude and direction of mid-parent heterosis varied considerably among the measured parameters and hybrid combinations.

Among the parameters for which higher values are generally desirable, Jmax exhibited the highest average mid-parent heterosis, at 54.57%, followed by CE and Vcmax, at 51.47% and 42.72%, respectively. The mid-parent heterosis of CE ranged from −67.22% to 259.06%, that of Vcmax ranged from −34.03% to 138.04%, and that of Jmax ranged from −21.33% to 293.09%. These broad ranges indicated substantial variation in the expression of heterosis among the 36 hybrid combinations.

The average mid-parent heterosis values of AQE and LSP were −9.27% and −1.75%, respectively, indicating that the F_1_ hybrids had slightly lower values than the parental means on average. The average mid-parent heterosis values of Rd and LCP were 17.62% and 12.67%, respectively, indicating that these parameters were generally higher in the F_1_ hybrids than in the corresponding parental means.

For the CO_2_-response parameters for which lower values may be physiologically favorable, Rp and CCP exhibited average mid-parent heterosis values of −25.53% and −35.65%, respectively. The mid-parent heterosis of Rp ranged from −88.59% to 75.17%, whereas that of CCP ranged from −88.96% to 239.75%. Thus, although both traits showed negative average mid-parent heterosis, their responses varied widely among individual hybrid combinations. The negative mean values indicated that the F_1_ hybrids generally had lower photorespiration rates and CO_2_ compensation points than the means of their corresponding parents.

The high-parent heterosis pattern is shown in **Figure 3B**. CE, Vcmax, and Jmax exhibited positive average high-parent heterosis values of 29.28%, 26.41%, and 17.08%, respectively. These results indicated that, on average, the F_1_ hybrids exceeded their better-performing parents for carboxylation efficiency, maximum Rubisco carboxylation rate, and maximum electron transport rate.

The average high-parent heterosis values of AQE and LSP were −11.57% and −15.73%, respectively, indicating that the F_1_ hybrids generally did not exceed the higher parental values for these two traits. The average high-parent heterosis values of Rd and LCP were −4.42% and −5.17%, respectively. Because lower Rd and LCP values may be favorable, these negative values indicated modest reductions in respiratory carbon loss and the minimum light requirement for positive net photosynthesis relative to the higher-valued parent.

The average high-parent heterosis values of Rp and CCP were −47.85% and −45.17%, respectively. These large negative values indicated that the F_1_ hybrids generally had substantially lower photorespiration rates and CO_2_ compensation points than the higher-valued parent. Overall, positive heterosis was most evident for CE, Vcmax, and Jmax, whereas negative heterosis was most pronounced for Rp and CCP. The wide ranges observed for these parameters also demonstrated considerable variation in heterosis among the different hybrid combinations.

### 3.7 Analysis of variance and general combining ability of light- and CO_2_-response parameters

The analysis of variance showed that block effects were not significant for any of the nine response-curve parameters (**Table 4**). In contrast, the effects of hybrid combination, maternal parent, paternal parent, and maternal parent × paternal parent interaction were significant or highly significant for all parameters. These results indicated that both additive parental effects and non-additive interaction effects contributed to the variation in photosynthetic traits among the F_1_ hybrids.

**Table 4:** Analysis of variance for light- and CO_2_-response parameters in the 36 F1 hybrid combinations.

Source	df	AQE	Rd	LSP	LCP	CE	Rp	CCP	Vcmax	Jmax
Block	2	1.45	0.02	0.38	1.44	0.69	0.57	2.72	0.13	0.70
Hybrid combination	35	27.01*	296.34**	231.47**	392.61**	979.57**	827.27**	773.99**	235.81**	145.07**
Maternal parent	5	5.83**	5.046**	7.19**	2.98*	6.67**	10.58**	2.88**	3.12**	8.12**
Paternal parent	5	6.32**	3.23*	4.47**	0.88*	7.32**	10.42**	4.29**	3.59**	7.99**
Maternal parent × paternal parent	25	27.41**	167.66**	245.45**	331.84**	824.48**	986.26**	596.30**	179.12**	143.69**

* and ** indicate significance at P < 0.05 and P < 0.01, respectively.

The general combining ability (GCA) effects varied among parental lines and traits (**Table 5**). Positive GCA effects were considered favorable for AQE, LSP, CE, Vcmax, and Jmax, whereas negative effects were considered favorable for Rd, LCP, Rp, and CCP. Among the maternal parents, TAM428A showed favorable GCA effects for AQE, CE, Vcmax, and Jmax; I15A performed well for LSP, Rd, and LCP; and L407A showed favorable effects for Rp and CCP. Among the paternal parents, JiR107 performed well for CE, Vcmax, and Rd; T40 showed favorable effects for LCP, Rp, and CCP; and Ji318R, 0-30, and Nan133 performed well for AQE, LSP, and Jmax, respectively. Overall, no single parental line exhibited favorable GCA effects for all parameters. However, TAM428A, I15A, and L407A among the maternal parents and JiR107, T40, and Ji318R among the paternal parents showed complementary advantages and may serve as useful parental materials for improving the photosynthetic performance of hybrid sorghum.

**Table 5:** General combining ability effects of the maternal and paternal parents for light- and CO_2_-response parameters.

Parent		AQE	Rd	LSP	LCP	CE	Rp	CCP	Vcmax	Jmax
Maternal parent	Ji2055A	-8.26	1.11	1.56	5.25	-31.76	15.96	41.13	-19.54	1.04
	TAM428A	6.54	-8.40	-3.08	-2.96	32.84	20.86	3.46	25.54	7.57
	521A	-3.32	18.54	-9.44	9.64	1.53	-8.44	-12.05	-3.69	1.34
	I15A	4.67	-30.71	12.42	-13.78	18.38	-5.74	-23.48	10.25	3.24
	170A	0.28	15.00	3.01	5.96	-17.45	6.73	21.66	-12.78	-9.70
	L407A	-0.04	3.39	-4.89	-4.37	-3.84	-29.32	-30.50	0.02	-3.72
Paternal parent	Nan133	2.44	8.22	4.96	7.09	14.80	2.49	-22.87	7.78	7.80
	Ji318R	3.91	-9.15	-7.09	-12.06	-5.16	19.99	8.86	-4.25	5.82
	T40	3.02	-13.88	-14.61	-12.66	2.53	-39.87	-45.96	-3.25	-9.33
	0-30	-13.36	36.83	22.42	29.43	-31.49	2.19	62.09	-15.99	-2.76
	501R	1.77	-1.85	-4.83	-7.54	-6.89	20.89	28.10	-9.41	2.09
	JiR107	2.15	-21.67	0.58	-4.23	27.08	-6.15	-31.91	24.61	-3.71

### 3.8 Specific combining ability of light- and CO_2_-response parameters

The specific combining ability (SCA) effects varied considerably among the 36 F_1_ hybrid combinations and among the nine response-curve parameters (**Supplementary Table 2**), indicating substantial differences in non-additive genetic effects among crosses. Positive SCA effects were considered favorable for AQE, LSP, CE, Vcmax, and Jmax, whereas negative effects were considered favorable for Rd, LCP, Rp, and CCP. Several hybrid combinations showed favorable SCA effects for multiple traits. Ji2055A × T40 performed well for AQE, Rd, LCP, Rp, CCP, and Jmax. TAM428A × 0–30 showed favorable effects for AQE, Rd, and LCP, whereas TAM428A × T40 performed well for CE and Vcmax. I15A × 0–30 exhibited favorable effects for AQE, Rd, LSP, LCP, Rp, CCP, and Jmax. In addition, 521A × JiR107 showed favorable effects for Rd, LSP, LCP, Rp, and CCP, while L407A × 0–30 performed well for AQE, CE, CCP, and Vcmax. The combination 170A × Ji318R had the highest favorable SCA effect for Jmax. Overall, no hybrid combination exhibited favorable SCA effects for all nine parameters. However, Ji2055A × T40, TAM428A × T40, TAM428A × 0-30, I15A × 0-30, 521A × JiR107, and L407A × 0–30 showed complementary advantages and may be useful combinations for improving the photosynthetic performance of hybrid sorghum.

### 3.9 Variance components and heritability of light- and CO_2_-response parameters

The variance components and heritability estimates of the nine light- and CO_2_-response parameters are presented in **Table 6**. Except for Rd, specific combining ability (SCA) accounted for more than 70% of the total combining-ability variance for all parameters, indicating that non-additive genetic effects made the major contribution to their genetic variation. The SCA proportions were particularly high for Rp, Jmax, LSP, and AQE, reaching 100.00%, 98.69%, 97.55%, and 96.26%, respectively. For Rd, the proportions of general combining ability (GCA) and SCA were 50.52% and 49.48%, respectively, suggesting that additive and non-additive effects contributed almost equally to this trait.

**Table 6:** Variance components and heritability estimates for light- and CO_2_-response parameters in hybrid sorghum.

Traits	Variance component				Variance ratio(%)		Heritability(%)	
	P_1_GCA	P_2_GCA	P_12_GCA	Env	GCA	SCA	H^2B^	H^2N^
AQE	0.00	0.03	0.71	0.09	3.74	96.26	89.34	3.34
R_d_	0.35	0.20	0.55	0.01	50.52	49.48	99.06	50.04
LSP	786.09	0.00	2851.48	404.17	2.45	97.55	98.71	2.42
LCP	36.49	0.00	118.98	1.14	23.47	76.53	99.27	23.30
CE	0.00	0.00	0.00	0.00	22.43	77.57	99.70	22.37
R_p_	0.00	0.00	0.11	0.00	0.00	100.00	99.69	0.00
CCP	0.77	0.12	2.57	0.01	25.60	74.40	99.59	25.50
Vcmax	131.09	186.72	756.19	13.29	29.59	70.41	98.78	29.23
Jmax	12.07	0.00	910.43	19.19	1.31	98.69	97.96	1.28

P_1_GCA, P_2_GCA, P_12_GCA, Env, GCA, SCA, H^2B^, H^2N^ represent the maternal-parent GCA variance, paternal-parent GCA variance, maternal × paternal interaction variance, environmental variance, proportion of GCA variance, proportion of SCA variance, broad-sense heritability, and narrow-sense heritability, respectively.

Broad-sense heritability exceeded 89% for all parameters, ranging from 89.34% for AQE to 99.70% for CE. In contrast, narrow-sense heritability was generally low. Rd had the highest narrow-sense heritability at 50.04%, followed by Vcmax, CCP, LCP, and CE, with values of 29.23%, 25.50%, 23.30%, and 22.37%, respectively. The narrow-sense heritability values of AQE, LSP, Rp, and Jmax were below 5%.

### 3.10 Heterotic grouping and relationships between photosynthetic traits and yield

**Figure 4:**
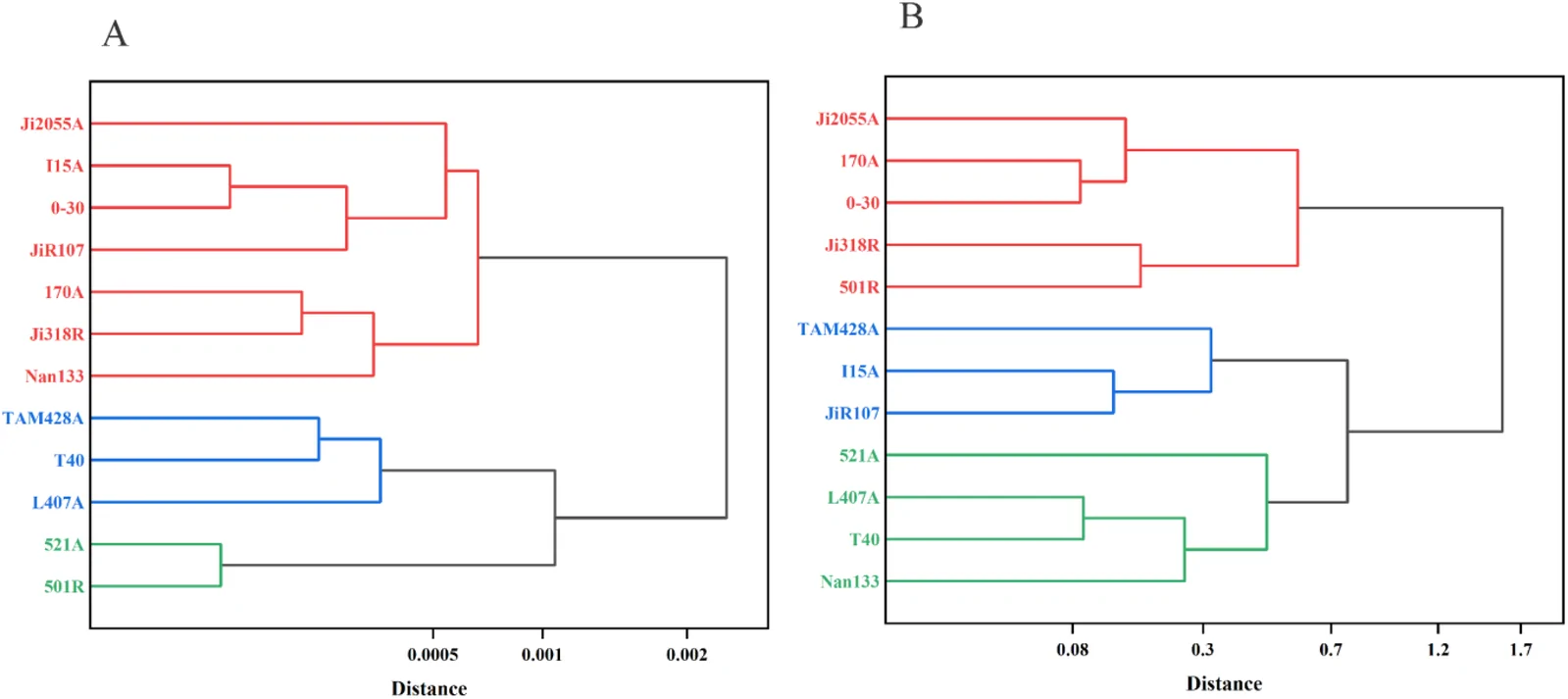
Cluster analysis of the 12 parental lines based on **(A)** phenotypic values of the nine light- and CO_2_-response parameters and **(B)** general combining ability effects.

Cluster analysis based on the nine light- and CO_2_-response parameters divided the 12 parental lines into three groups at the selected Euclidean distance of 0.001 (**Figure 4A**). Group I consisted of Ji2055A, I15A, 0-30, JiR107, 170A, Ji318R, and Nan133; Group II consisted of TAM428A, T40, and L407A; and Group III consisted of 521A and 501R. Clustering based on the GCA effects also produced three groups (**Figure 4B**). Group I included Ji2055A, 170A, 0-30, Ji318R, and 501R; Group II included TAM428A, I15A, and JiR107; and Group III included 521A, L407A, T40, and Nan133. The differences between the two clustering results indicated that parental phenotypic performance and GCA effects provided complementary information for heterotic-group classification.

**Figure 5:**
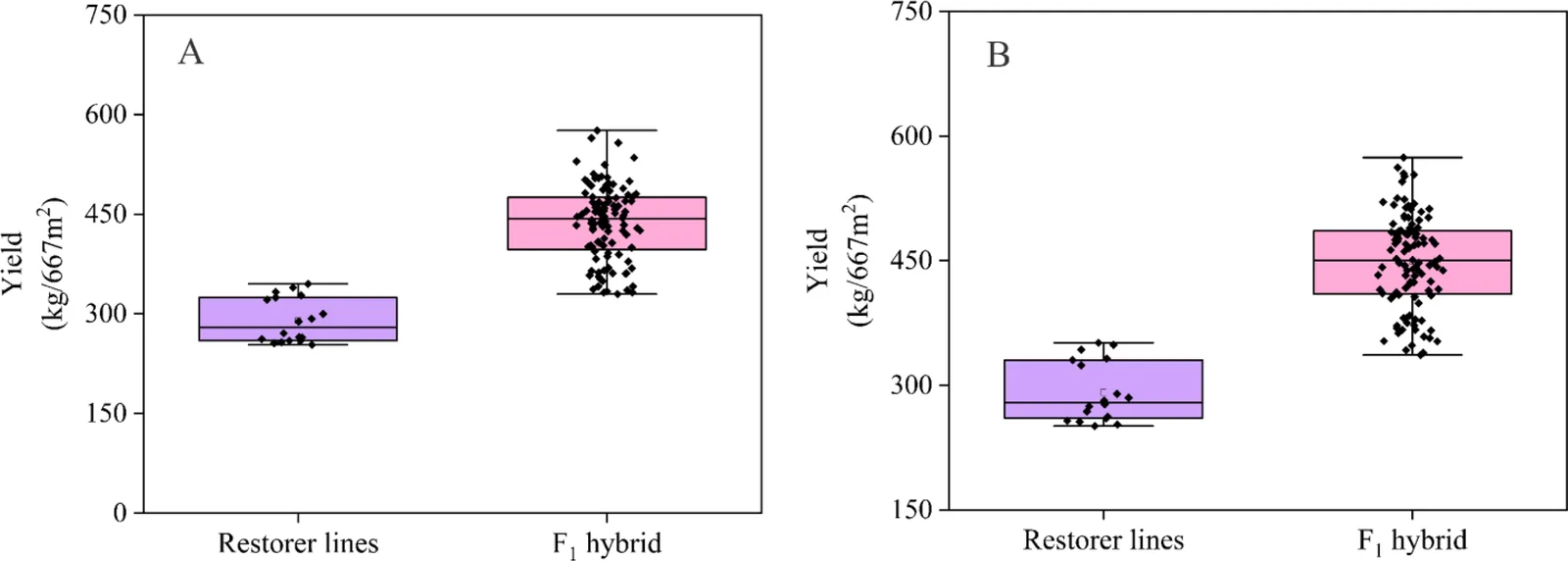
Grain yield of the restorer lines and F_1_ hybrids in **(A)** 2021 and **(B)** 2022.

The F_1_ hybrids had higher mean grain yield than the restorer lines in both experimental years (**Figure 5**). In 2021, the mean yields of the restorer lines and F_1_ hybrids were 290.03 and 436.61 kg per 667 m², respectively, representing an increase of 50.54%. In 2022, the corresponding yields were 291.42 and 449.09 kg per 667 m², representing an increase of 54.10%.

**Figure 6:**
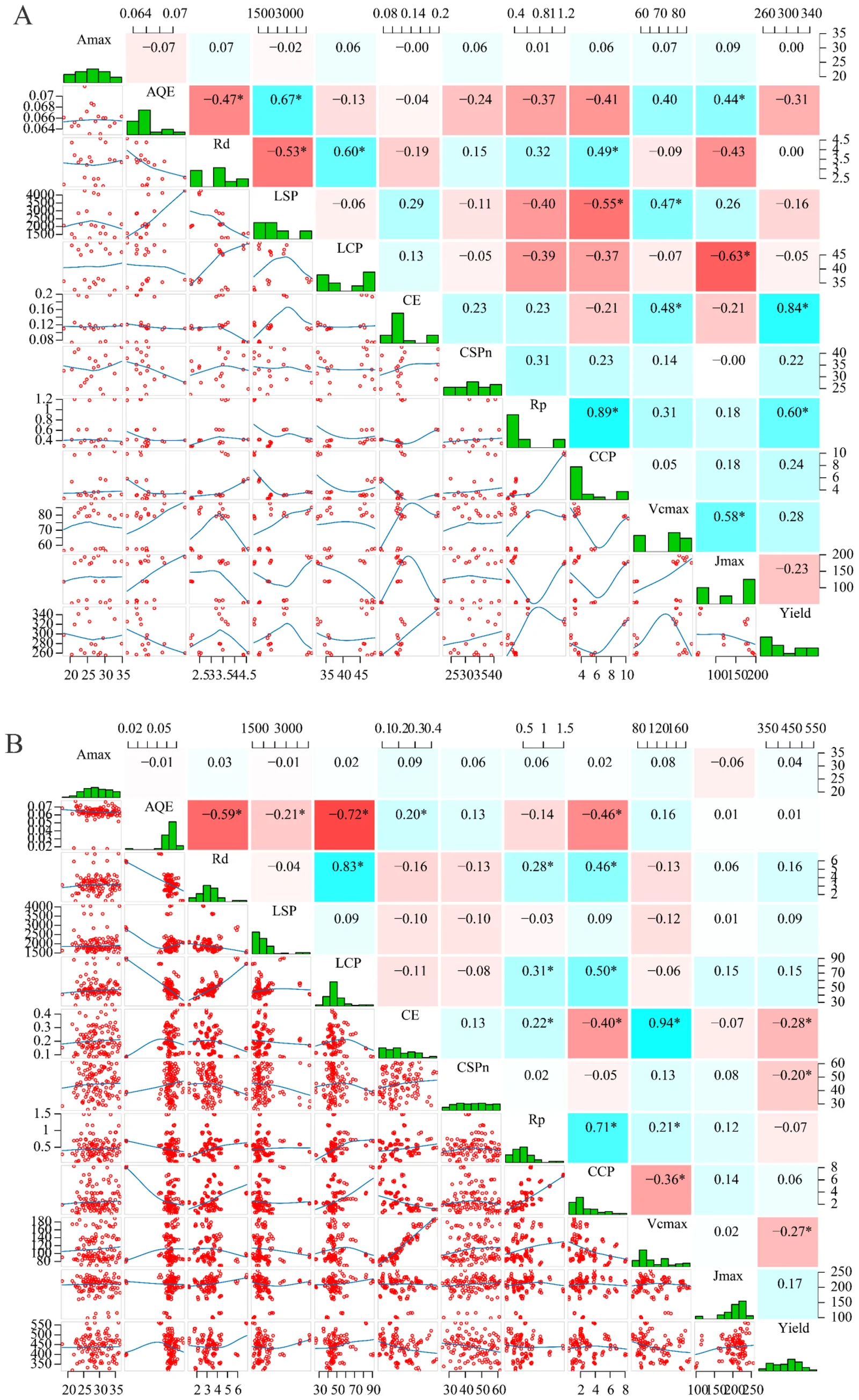
Correlations between yield and photosynthetic response parameters in **(A)** the restorer lines and **(B)** the F_1_ hybrids.

The relationships between yield and the photosynthetic response parameters differed between the restorer lines and F_1_ hybrids (**Figure 6**). In the restorer lines, yield was significantly and positively correlated with CE and Rp. In the F_1_ hybrids, yield was significantly and negatively correlated with CE, CSPn, and Vcmax. These contrasting correlation patterns indicated differences in trait–yield relationships between the two genotype groups but did not establish causal relationships between individual photosynthetic parameters and yield.

**Figure 7:**
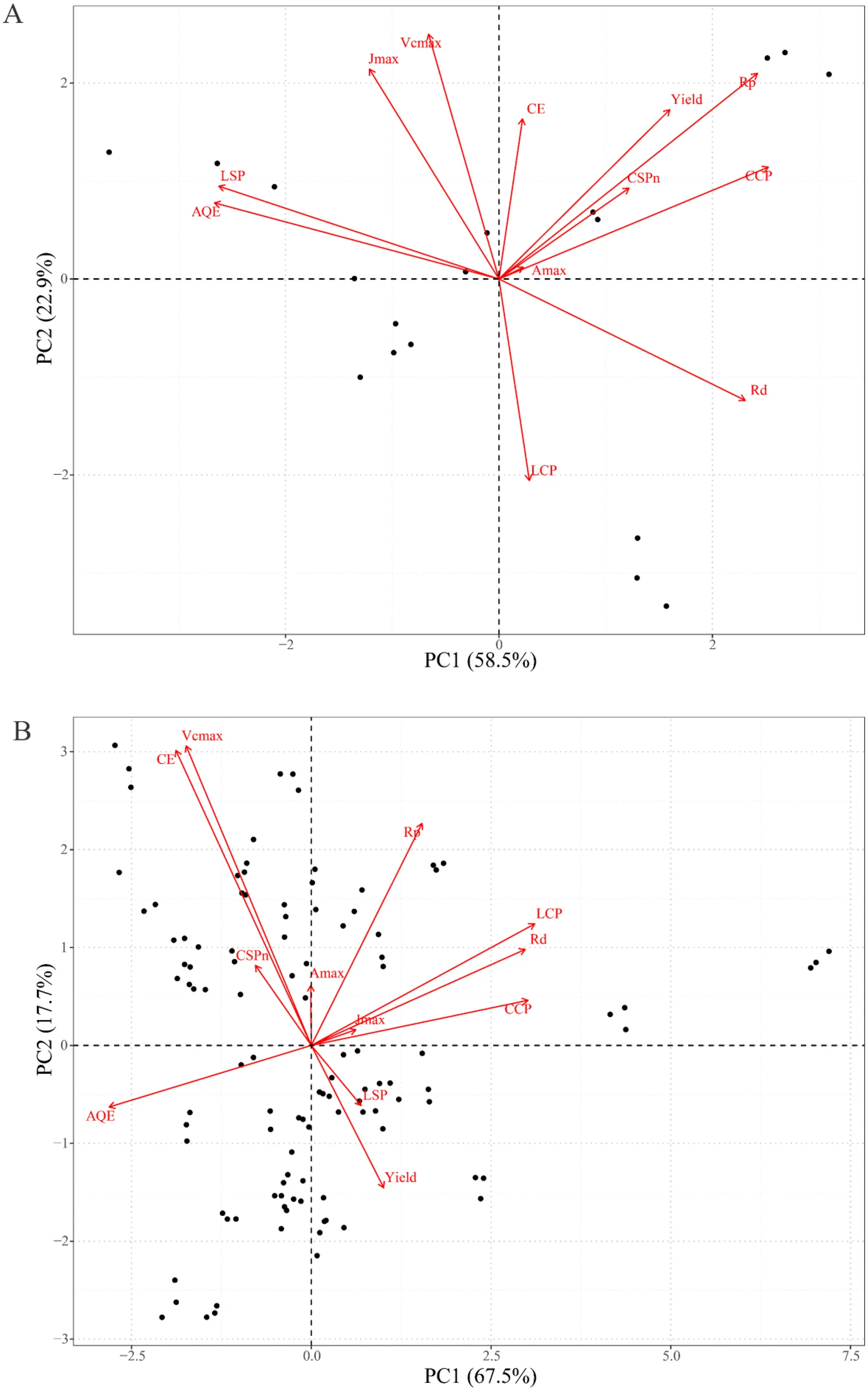
Principal component analysis of yield and photosynthetic response parameters in **(A)** the restorer lines and **(B)** the F_1_ hybrids.

Principal component analysis further revealed different multivariate patterns between the restorer lines and F_1_ hybrids (**Figure 7**). In the restorer lines, the first two principal components explained 81.4% of the total variation, with PC1 and PC2 accounting for 58.5% and 22.9%, respectively. PC1 was mainly associated with Rp, CCP, Rd, and yield, whereas PC2 was mainly associated with Vcmax and Jmax. In the F_1_ hybrids, the first two principal components explained 85.2% of the total variation, with PC1 and PC2 accounting for 67.5% and 17.7%, respectively. PC1 was mainly associated with LCP, CCP, and Rd, whereas PC2 was mainly associated with Vcmax and CE. These results demonstrated that the major sources of trait variation differed between the restorer lines and F_1_ hybrids.

## 4 Discussion

### 4.1 Stage-dependent changes in leaf light- and CO_2_-response characteristics

The light- and CO_2_-response characteristics differed among growth stages and genotype groups, indicating that the photosynthetic performance of the F_1_ hybrids was developmentally dependent rather than consistently superior throughout the entire growth period. At the jointing stage, the F_1_ hybrids had lower AQE than both parental groups, but their Amax was comparable to that of the maternal parents and higher than that of the paternal parents. At the flowering stage, the F_1_ hybrids exhibited the highest Amax and CSPn, together with relatively high Vcmax and Jmax. However, these advantages were not maintained at maturity, when the F_1_ hybrids had lower Amax, LSP, CE, Vcmax, and Jmax than the paternal parents. Previous research has also shown that sorghum hybrids may express heterosis in photosynthetic traits at flowering, supporting the conclusion that the expression of photosynthetic advantages is closely associated with developmental stage (Zhao et al., 2024).

The fitted light-response parameters further demonstrated that individual photosynthetic traits did not change in parallel. At the jointing stage, the lower AQE of the F_1_ hybrids indicated weaker initial light-use efficiency under low irradiance, whereas their relatively high Amax suggested that this limitation did not prevent them from maintaining a high photosynthetic rate under light-saturated conditions. At flowering, the F_1_ hybrids combined the highest Amax with a lower LCP than both parental groups, indicating favorable photosynthetic performance under both low- and high-light conditions. At maturity, the F_1_ hybrids retained the lowest Rd, but their Amax and LSP were also the lowest among the three genotype groups. Thus, reduced respiratory carbon loss alone was insufficient to maintain high photosynthetic capacity during the late growth stage. Natural variation in sorghum photosynthetic responses is strongly influenced by genotype and environmental conditions, and the responses of light-use traits may differ among materials and measurement environments (Ortiz and Salas-Fernandez, 2022; Yang et al., 2024).

The CO_2_-response parameters showed a similar stage-dependent pattern. At the jointing and flowering stages, the F_1_ hybrids exhibited higher CSPn, Vcmax, and Jmax and lower CCP than both parental groups. These results suggested that their photosynthetic advantages at these stages were primarily associated with greater CO_2_-saturated assimilation capacity, Rubisco carboxylation capacity, and electron-transport capacity, together with a lower CO_2_ requirement for achieving positive net photosynthesis. In contrast, CE was not consistently higher in the F_1_ hybrids: it was lower than that of the maternal parents at jointing and did not differ significantly from the maternal parents at flowering. Therefore, the enhanced CO_2_-response capacity of the F_1_ hybrids could not be attributed solely to greater initial carboxylation efficiency. At maturity, the F_1_ hybrids no longer differed from the parental groups in CSPn or Rp and had lower CE, Vcmax, and Jmax than the paternal parents, confirming that their CO_2_-assimilation advantage weakened during the late growth stage.

Overall, the F_1_ hybrids displayed their clearest photosynthetic advantages during the jointing and flowering stages, particularly in light-saturated photosynthetic capacity, CO_2_-saturated assimilation capacity, Rubisco carboxylation capacity, and electron transport. However, the direction and magnitude of these advantages varied among parameters and were not maintained at maturity. These findings emphasize that the photosynthetic performance of hybrid sorghum should be evaluated using multiple response-curve parameters and at multiple developmental stages. Measurements conducted at only one growth stage or based on a single photosynthetic parameter may provide an incomplete assessment of hybrid performance. The favorable leaf-level characteristics observed during the jointing and flowering stages may contribute to biomass accumulation and yield formation, but their relationships with yield should be interpreted as associations rather than direct causal effects.

### 4.2 Heterosis, combining ability, and breeding implications of light- and CO_2_-response parameters

The heterosis of the light- and CO_2_-response parameters varied substantially among traits and hybrid combinations. CE, Vcmax, and Jmax exhibited positive average mid-parent heterosis of 51.47%, 42.72%, and 54.57%, respectively, and positive average high-parent heterosis of 29.28%, 26.41%, and 17.08%, respectively. These results indicated that many F_1_ hybrids had greater carboxylation efficiency, Rubisco carboxylation capacity, and electron-transport capacity than their parental references. Previous studies have also reported trait- and cross-dependent heterosis in sorghum photosynthetic and agronomic characteristics, emphasizing that the expression of heterosis differs among physiological processes and parental combinations (Zhao et al., 2024; Zhang et al., 2024).

In contrast, Rp and CCP exhibited negative average mid-parent heterosis of −25.53% and −35.65%, respectively, and negative average high-parent heterosis of −47.85% and −45.17%, respectively. Because lower Rp and CCP values may indicate reduced photorespiratory carbon loss and a lower CO_2_ requirement for maintaining positive net photosynthesis, these reductions may be physiologically favorable. However, the negative values should be interpreted according to the biological direction of each trait rather than being described simply as negative or inferior heterosis. In addition, high-parent heterosis was calculated relative to the higher-valued parent; therefore, a negative value does not necessarily demonstrate superiority over the physiologically more favorable lower-valued parent. The wide ranges of heterosis observed for CE, Rp, CCP, Vcmax, and Jmax further indicated that the photosynthetic performance of a hybrid could not be predicted solely from the phenotypic values of its parents.

The GCA effects also varied among parental lines and traits. No parent showed favorable GCA effects for all nine response parameters, indicating that the genetic contributions of the parental lines were trait specific. Among the maternal parents, TAM428A showed favorable effects mainly for AQE, CE, Vcmax, and Jmax, whereas I15A and L407A showed favorable effects for different components of light utilization and respiratory or photorespiratory carbon loss. Among the paternal parents, JiR107 showed favorable effects for CE, Vcmax, and Rd, whereas T40 showed favorable effects for LCP, Rp, and CCP. This complementarity suggests that parental selection should be based on clearly defined breeding objectives rather than on an overall ranking across all photosynthetic parameters.

The SCA effects differed markedly among hybrid combinations, and several crosses showed favorable effects for multiple traits. Ji2055A × T40, TAM428A × T40, TAM428A × 0-30, I15A × 0-30, 521A × JiR107, and L407A × 0–30 were among the combinations with complementary SCA advantages. Nevertheless, high GCA in a parent did not consistently result in high SCA in all of its hybrids. This result is consistent with the distinction between GCA, which primarily represents additive parental effects, and SCA, which reflects deviations associated mainly with dominance and epistatic interactions (Griffing, 1956). Similar trait-dependent differences in GCA, SCA, and heterosis have been reported in sorghum, supporting the combined evaluation of parental combining ability and individual hybrid performance when identifying promising crosses (Zhang et al., 2024; Volpato et al., 2024).

The variance-component analysis provided further evidence for the importance of non-additive genetic effects. Except for Rd, SCA accounted for more than 70% of the total combining-ability variance for all response parameters. The SCA proportions were particularly high for Rp, Jmax, LSP, and AQE. In contrast, the GCA and SCA proportions for Rd were 50.52% and 49.48%, respectively, indicating that additive and non-additive effects contributed almost equally to the variation in this trait. These results suggested that the performance of most response parameters depended strongly on the specific combinations of parental alleles rather than solely on the average effects transmitted by individual parents.

Broad-sense heritability exceeded 89% for all nine parameters, whereas narrow-sense heritability was generally low. This contrast is important because broad-sense heritability includes additive, dominance, and epistatic genetic variation, whereas narrow-sense heritability represents only the additive component that can be predictably transmitted through parental selection. Therefore, the high broad-sense heritability observed in this study should not be interpreted as evidence that all traits can be efficiently improved through direct phenotypic selection of parental lines. In particular, the narrow-sense heritability values of AQE, LSP, Rp, and Jmax were below 5%, suggesting limited efficiency of parental phenotypic selection for these parameters. For such traits, replicated evaluation of specific F_1_ combinations may be more informative than selection based solely on parental performance. By comparison, the relatively high narrow-sense heritability of Rd, at 50.04%, suggests greater potential for improvement through selection of parents with favorable additive effects.

Photosynthetic traits are also sensitive to genotype, developmental stage, and measurement environment. Substantial genetic variation has been reported for sorghum gas-exchange and photosynthetic traits under contrasting environmental conditions, but their expression may change with water availability and other environmental factors (Ortiz and Salas-Fernandez, 2022; Amombo et al., 2024; Al-Salman et al., 2024). Consequently, the variance components and heritability estimates obtained in the present experiment should be interpreted within the tested genetic materials and field conditions. Multi-environment evaluation and prediction models that account for both additive and dominance effects may improve the reliability of hybrid selection and the prediction of sorghum hybrid performance (Maulana et al., 2023; Volpato et al., 2024).

The phenotypic and GCA-based clustering analyses both separated the 12 parental lines into three groups, but the composition of the groups differed. This discrepancy indicated that similarity in parental photosynthetic performance did not necessarily correspond to similarity in combining ability. Phenotypic clustering described the observed performance of the parents, whereas GCA-based clustering reflected their average genetic contributions to hybrid progeny. The two approaches therefore provided complementary information. However, because the classification was based on only 12 parental lines and has not yet been independently validated using the yield performance of crosses between groups, the proposed heterotic groups should be regarded as preliminary rather than definitive.

The F_1_ hybrids had mean grain yields that were 50.54% and 54.10% higher than those of the restorer lines in 2021 and 2022, respectively. These differences demonstrated a consistent yield advantage of the hybrids over the restorer-line group. Nevertheless, they should not be described as statistically significant yield heterosis unless an explicit heterosis test is performed using the appropriate parental reference. Previous studies have demonstrated that sorghum hybrid yield is influenced by both combining ability and the performance of individual crosses and should be evaluated across environments before superior combinations are selected (Maulana et al., 2023; Volpato et al., 2024; Zhang et al., 2024).

The contrasting correlations between photosynthetic parameters and yield in the restorer lines and F_1_ hybrids should also be interpreted cautiously. In the restorer lines, yield was positively correlated with CE and Rp, whereas in the F_1_ hybrids, yield was negatively correlated with CE, CSPn, and Vcmax. These relationships did not demonstrate that hybridization altered the physiological basis of yield formation or that higher CE, CSPn, or Vcmax directly reduced hybrid yield. Alternative explanations include saturation of leaf-level photosynthetic capacity, differences in phenology and canopy architecture, variation in source–sink coordination, or differences in yield components such as panicle size, grain number, and grain weight. The unequal numbers of restorer lines and F_1_ hybrids may also have affected the stability of the correlation estimates. Therefore, these correlations should be regarded as exploratory associations that require validation using larger populations and direct measurements of biomass accumulation, canopy photosynthesis, assimilate partitioning, and yield components.

Overall, the results support a breeding strategy that combines parental GCA, cross-specific SCA, stage-specific photosynthetic evaluation, and multi-environment yield testing. Parental GCA may assist in selecting materials with favorable additive effects for specific traits, whereas direct evaluation of hybrid combinations is essential for parameters predominantly controlled by non-additive effects. Photosynthetic response parameters may provide useful supplementary criteria for hybrid selection, but they should be evaluated jointly with yield and source–sink traits rather than used as independent predictors of yield performance.

## 5 Conclusion

The light- and CO_2_-response characteristics of the F_1_ hybrids varied with growth stage. Their photosynthetic advantages were mainly expressed at the jointing and flowering stages, when the hybrids generally showed higher light- or CO_2_-saturated photosynthetic capacity, Rubisco carboxylation capacity, and electron-transport capacity, together with relatively low respiratory or photorespiratory carbon loss. However, these advantages were not consistently maintained at maturity, indicating that hybrid photosynthetic performance should be evaluated across multiple developmental stages. CE, Vcmax, and Jmax showed positive average mid-parent and high-parent heterosis, whereas Rp and CCP showed negative average heterosis. The magnitude and direction of heterosis differed among traits and hybrid combinations. TAM428A, I15A, and L407A among the maternal parents and JiR107, T40, and Ji318R among the paternal parents showed complementary GCA advantages, while several crosses exhibited favorable SCA effects for multiple traits. Except for Rd, SCA accounted for more than 70% of the total combining-ability variance for all response parameters. Broad-sense heritability exceeded 89%, whereas narrow-sense heritability was generally low, indicating that most traits were mainly influenced by non-additive genetic effects. Therefore, direct selection based only on parental phenotypic performance may be inefficient, and evaluation of specific hybrid combinations is essential. The F_1_ hybrids had mean grain yields 50.54% and 54.10% higher than those of the restorer lines in 2021 and 2022, respectively. Overall, integrating stage-specific photosynthetic evaluation, parental GCA, cross-specific SCA, and multi-environment yield testing may improve the selection of promising sorghum parents and hybrids.

## Data Availability

The original contributions presented in the study are included in the article/**Supplementary Material**. Further inquiries can be directed to the corresponding author.
