# Supplementary material for: Adaptability and production potential of sorghum in cool regions: heterosis analysis of parameters of light response and CO2 response curves
Source: Front Plant Sci. 2026 Aug 19;17:1905153. doi: 10.3389/fpls.2026.1905153 (PMC13534009; doi:10.3389/fpls.2026.1905153)
Supplement: Supplementary file 1 [file Table1.docx]

Supplementary Material

Table S1 Differences in light and CO_2_ response curve parameters of sorghum leaves

| Genotypes | | AQE | R_d_ | LSP | LCP | CE | Rp | CCP | Vcmax | Jmax |
| --- | --- | --- | --- | --- | --- | --- | --- | --- | --- | --- |
|  |  |  | (μmol·m^-2^·s^-1^) | (μmol·m^-2^·s^-1^) | (μmol·m^-2^·s^-1^) |  | (μmol·m^-2^·s^-1^) | (μmol·m^-2^·s^-1^) | (μmol·m^-2^·s^-1^) | (μmol·m^-2^·s^-1^) |
| Male sterile | Ji2055A | 0.0683 | 4.16 | 2875.04 | 59.98 | 0.402 | 0.847 | 2.136 | 167.05 | 177.41 |
| lines | TAM428A | 0.0678 | 1.73 | 2087.71 | 20.17 | 0.249 | 1.552 | 6.371 | 108.11 | 167.08 |
|  | 521A | 0.0676 | 3.17 | 1623.81 | 49.11 | 0.165 | 0.53 | 3.312 | 96.33 | 212.44 |
|  | I15A | 0.058 | 1.49 | 2040.03 | 53.57 | 0.079 | 1.037 | 6.344 | 62.03 | 140.1 |
|  | 170A | 0.0664 | 3.33 | 1657.94 | 68.2 | 0.095 | 0.392 | 4.179 | 43.56 | 69.84 |
|  | L407A | 0.0691 | 2.97 | 1668.89 | 47.17 | 0.138 | 1.116 | 8.194 | 85.63 | 174.9 |
| Restorer | Nan133 | 0.0646 | 3.34 | 2850.14 | 46.88 | 0.198 | 0.61 | 3.177 | 81.62 | 60.33 |
| lines | Ji318R | 0.066 | 4.38 | 1542.29 | 47.11 | 0.077 | 0.423 | 5.619 | 56.17 | 54.61 |
|  | T40 | 0.0658 | 3.49 | 2167.4 | 44.99 | 0.116 | 0.363 | 3.136 | 84.11 | 182.74 |
|  | 0-30 | 0.066 | 2.11 | 1977.56 | 36.03 | 0.112 | 0.289 | 2.589 | 58.02 | 119.77 |
|  | 501R | 0.0631 | 3.61 | 1258.62 | 33.41 | 0.121 | 1.182 | 9.889 | 79.51 | 171.9 |
|  | JiR107 | 0.0684 | 2.48 | 4139.21 | 35.3 | 0.108 | 0.391 | 3.637 | 83.12 | 190.7 |
| F1 hybrid | Ji2055A×Nan133 | 0.0666 | 3.01 | 1685.04 | 43.11 | 0.157 | 0.295 | 1.884 | 88.79 | 211.95 |
|  | Ji2055A×Ji318R | 0.0651 | 2.74 | 2219.42 | 44.63 | 0.155 | 0.275 | 1.784 | 81.92 | 190.94 |
|  | Ji2055A×T40 | 0.0683 | 2.11 | 1682.64 | 37 | 0.101 | 0.069 | 0.686 | 82.84 | 206.24 |
|  | Ji2055A×0-30 | 0.0183 | 5.79 | 2845.16 | 91.17 | 0.084 | 0.674 | 8.027 | 81.85 | 218.52 |
|  | Ji2055A×501R | 0.0619 | 3.65 | 1847.08 | 47.11 | 0.221 | 1.493 | 6.885 | 123.57 | 186.24 |
|  | Ji2055A×JiR107 | 0.0674 | 2.81 | 2138.52 | 47.42 | 0.153 | 0.577 | 3.799 | 93.22 | 217.41 |
|  | TAM428A×Nan133 | 0.0697 | 3.92 | 1887.04 | 68.09 | 0.25 | 0.722 | 2.914 | 135.46 | 231.13 |
|  | TAM428A×Ji318R | 0.0669 | 2.94 | 1780.59 | 41.65 | 0.238 | 1.153 | 4.913 | 134.16 | 233.86 |
|  | TAM428A×T40 | 0.068 | 2.93 | 1781.28 | 44.68 | 0.416 | 0.484 | 1.172 | 182.61 | 196.78 |
|  | TAM428A×0-30 | 0.0701 | 3.27 | 2061.09 | 45.84 | 0.104 | 0.625 | 6.06 | 89.49 | 222.75 |
|  | TAM428A×501R | 0.0689 | 3.22 | 2155.14 | 43.91 | 0.284 | 0.254 | 0.898 | 135.77 | 213.84 |
|  | TAM428A×JiR107 | 0.0613 | 1.84 | 2070.53 | 41.39 | 0.319 | 0.315 | 0.991 | 169.7 | 215.71 |
|  | 521A×Nan133 | 0.0584 | 3.82 | 1654.67 | 50.99 | 0.309 | 0.468 | 1.524 | 141.15 | 215.37 |
|  | 521A×Ji318R | 0.064 | 3.66 | 1599.01 | 44.35 | 0.307 | 0.57 | 1.874 | 142.84 | 227.48 |
|  | 521A×T40 | 0.0631 | 3.73 | 1800.73 | 47.88 | 0.114 | 0.115 | 1.011 | 84.33 | 198.1 |
|  | 521A×0-30 | 0.0543 | 6.8 | 1527.72 | 82.64 | 0.147 | 0.56 | 3.832 | 99.23 | 233.12 |
|  | 521A×501R | 0.0636 | 3.25 | 2178.13 | 51.54 | 0.166 | 0.925 | 5.757 | 74.98 | 191 |
|  | 521A×JiR107 | 0.0626 | 2.1 | 2320.73 | 45.69 | 0.211 | 0.096 | 0.454 | 109.18 | 166.35 |
|  | I15A×Nan133 | 0.0654 | 3.33 | 2083.57 | 47.54 | 0.386 | 0.744 | 1.953 | 170.98 | 204.41 |
|  | I15A×Ji318R | 0.0742 | 1.25 | 2108.71 | 26.82 | 0.114 | 0.263 | 2.306 | 85.8 | 199.35 |
|  | I15A×T40 | 0.0639 | 2.37 | 1713.02 | 45.75 | 0.264 | 0.367 | 1.394 | 127.63 | 199.93 |
|  | I15A×0-30 | 0.0648 | 2.58 | 4073.08 | 40.58 | 0.166 | 0.355 | 2.148 | 93.44 | 219.05 |
|  | I15A×501R | 0.0627 | 1.84 | 1795.92 | 34.8 | 0.19 | 0.136 | 2.148 | 105.16 | 220.16 |
|  | I15A×JiR107 | 0.06 | 2.6 | 1757.87 | 60.42 | 0.336 | 0.943 | 2.841 | 168.81 | 218.93 |
|  | 170A×Nan133 | 0.0612 | 3.52 | 3594.6 | 55.96 | 0.212 | 0.587 | 2.782 | 106.25 | 221.1 |
|  | 170A×Ji318R | 0.0653 | 4.33 | 1816.82 | 56.31 | 0.189 | 0.714 | 3.797 | 108.19 | 244.61 |
|  | 170A×T40 | 0.0631 | 3.51 | 1566.02 | 40.02 | 0.186 | 0.429 | 2.328 | 87.29 | 100.26 |
|  | 170A×0-30 | 0.0612 | 4.4 | 1766.88 | 61.02 | 0.087 | 0.458 | 5.296 | 69.64 | 172.17 |
|  | 170A×501R | 0.0632 | 3.71 | 1810.69 | 52.09 | 0.124 | 0.477 | 3.871 | 84.02 | 198.94 |
|  | 170A×JiR107 | 0.0612 | 2.93 | 1906.68 | 44.92 | 0.253 | 0.49 | 1.955 | 139.46 | 163.02 |
|  | L407A×Nan133 | 0.0633 | 3.4 | 1750.97 | 46.91 | 0.104 | 0.207 | 1.988 | 88.15 | 236.21 |
|  | L407A×Ji318R | 0.0605 | 3.03 | 1779.46 | 46.67 | 0.179 | 0.58 | 3.258 | 97.86 | 186.83 |
|  | L407A×T40 | 0.0582 | 2.58 | 1923.07 | 46.37 | 0.191 | 0.444 | 2.345 | 91.9 | 212.29 |
|  | L407A×0-30 | 0.0572 | 4.38 | 2355.34 | 56.49 | 0.272 | 0.32 | 1.183 | 143.98 | 115.91 |
|  | L407A×501R | 0.0639 | 3.57 | 1764.3 | 43.51 | 0.162 | 0.236 | 1.458 | 95.49 | 226.95 |
|  | L407A×JiR107 | 0.0692 | 3.13 | 1990.49 | 41.64 | 0.29 | 0.394 | 1.366 | 167.68 | 192.99 |
| Maternal-line mean | | 0.0662 | 2.81 | 1992.24 | 49.69 | 0.188 | 0.912 | 5.089 | 93.79 | 156.96 |
| R-lines mean | | 0.0657 | 3.23 | 2322.54 | 40.62 | 0.122 | 0.543 | 4.675 | 73.76 | 130.01 |
| Hybrids mean | | 0.0627 | 3.28 | 2022 | 49.08 | 0.207 | 0.495 | 2.747 | 113.41 | 203.05 |
| Mean | | 0.0649 | 3.11 | 2112.26 | 46.47 | 0.172 | 0.65 | 4.17 | 93.65 | 163.34 |
| SD | | 0.0076 | 0.68 | 293.67 | 9.27 | 0.049 | 0.141 | 0.377 | 11.17 | 15.45 |
| CV(%) | | 11.71 | 21.86 | 13.9 | 19.94 | 28.49 | 21.69 | 9.04 | 11.92 | 9.46 |
| Difference in combinations (F) | | 7.923** | 15.457** | 16.891** | 11.305** | 12.476** | 4.412* | 5.159** | 4.479* | 12.19** |

Table S2 Specific combining ability effects of light- and CO₂-response parameters in the 36 F₁ hybrid combinations

| Cross | AQE | R_d_ | LSP | LCP | CE | R_p_ | CCP | V_cmax_ | J_max_ |
| --- | --- | --- | --- | --- | --- | --- | --- | --- | --- |
| Ji2055A×Nan133 | 16.1 | -16.59 | -24.97 | -25.49 | -6.54 | -59.95 | -48.11 | -10.22 | -4.14 |
| Ji2055A×Ji318R | 4.88 | -12.28 | 12.7 | -2.66 | 9.42 | -77.82 | -93.68 | -5.12 | -12.4 |
| Ji2055A×T40 | 15.83 | -25.36 | -5.9 | -18.66 | -25.48 | -70.15 | -70.23 | -5.23 | 8.56 |
| Ji2055A×0-30 | -53.44 | 35.76 | 20.99 | 55.65 | 0.44 | 25.36 | 86.02 | 7.66 | 9.53 |
| Ji2055A×501R | 5.18 | 13.96 | -6.29 | -2.4 | 45.59 | 186.21 | 89.36 | 38.6 | -11.31 |
| Ji2055A×JiR107 | 13.4 | 4.19 | 3.18 | -4.71 | -24.75 | 6.03 | 31.75 | -24.59 | 9.96 |
| TAM428A×Nan133 | 0.21 | 21.12 | -11.95 | 39.74 | -25.39 | 25.17 | 23.99 | -13.25 | -3.01 |
| TAM428A×Ji318R | -3.94 | 8.23 | -0.59 | -1.17 | -8.45 | 94.94 | 70.99 | -2.71 | 2.21 |
| TAM428A×T40 | -4.28 | 9.5 | 7.46 | 4.26 | 70.52 | 14.11 | -15.79 | 40.35 | -2.24 |
| TAM428A×0-30 | 18.15 | -30.87 | -14.82 | -33.99 | -53.13 | 9.09 | 57.81 | -31.13 | 6.79 |
| TAM428A×501R | 2.71 | 8.04 | 13.89 | -0.09 | 16.44 | -88.1 | -100.17 | 4.12 | -4.48 |
| TAM428A×JiR107 | -13.56 | -16.09 | 6.95 | -7.95 | -2.36 | -54.2 | -40.49 | 1.88 | 0.9 |
| 521A×Nan133 | -6.89 | -10.96 | -14.29 | -13.21 | 35.4 | 0.66 | -13.28 | 23.09 | -3.93 |
| 521A×Ji318R | -2.26 | 1.68 | -7.11 | -10.19 | 54.19 | 3.58 | -28.89 | 39.25 | 7.42 |
| 521A×T40 | -0.2 | 8.47 | 11.41 | 0.41 | -49.61 | -34.85 | -4.35 | -20.39 | 5.24 |
| 521A×0-30 | 5.61 | 50.82 | -37.77 | 33.61 | 1.26 | 19.57 | -8.85 | 6.71 | 16.41 |
| 521A×501R | 0.47 | -18.34 | 22.41 | 2.21 | -15.62 | 81.92 | 96.36 | -21.2 | -9.25 |
| 521A×JiR107 | 3.11 | -32.1 | 24.84 | -12.32 | -25.47 | -72.84 | -41.21 | -27.1 | -15.88 |
| I15A×Nan133 | -5.79 | 23.37 | -14.28 | 3.12 | 58.85 | 55.05 | 15.6 | 30.59 | -10.64 |
| I15A×Ji318R | 9.82 | -23.75 | -0.39 | -20.32 | -58.38 | -61.57 | -2.65 | -31.98 | -12.18 |
| I15A×T40 | 0.94 | 12.28 | -14.41 | 17.82 | 7.84 | 16.4 | 19.81 | 7.34 | 3.1 |
| I15A×0-30 | 9.92 | -31.29 | 77.36 | -34.09 | -4.91 | -27.89 | -63.94 | -14.19 | 8.39 |
| I15A×501R | -5.47 | -14.04 | -17.51 | -7.9 | -20.92 | -94.96 | -29.8 | -7.56 | 4.43 |
| I15A×JiR107 | -9.93 | 34.28 | -26.57 | 41.58 | 21.17 | 107.46 | 58.52 | 14.72 | 6.76 |
| 170A×Nan133 | -5.88 | -12.38 | 76.03 | 1.38 | 1.38 | 13.07 | -0.72 | -0.89 | 11.01 |
| 170A×Ji318R | -1.18 | 30.25 | -5.69 | 23.73 | 11.08 | 21.2 | 6.24 | 12.19 | 24.31 |
| 170A×T40 | -1.53 | 6.35 | -12.05 | -13.34 | 2.48 | 14.51 | 10.26 | -6.48 | -34.01 |
| 170A×0-30 | 11.29 | -19.5 | -42.67 | -9.72 | -11.36 | -16.78 | 16.46 | -11.62 | -3.47 |
| 170A×501R | -0.37 | -1.16 | -10.09 | 9.19 | -15.89 | -27.78 | -12.7 | -4.48 | 4.87 |
| 170A×JiR107 | -2.84 | -4.24 | -7.09 | -10.84 | 12 | -3.88 | -20.21 | 11.69 | -4.65 |
| L407A×Nan133 | 2.37 | -4.29 | -13.35 | -5.29 | -64.02 | -34.51 | 22.25 | -30.12 | 10.73 |
| L407A×Ji318R | -7.31 | -3.69 | 1.3 | 11.68 | -6.92 | 22.02 | 40.79 | -10.37 | -9.93 |
| L407A×T40 | -10.76 | -10.87 | 14.84 | 10.08 | -5.93 | 58.6 | 66.61 | -17 | 18.74 |
| L407A×0-30 | 9.03 | -8.23 | 0.95 | -9.86 | 68.41 | -8.28 | -88.26 | 42.57 | -37.06 |
| L407A×501R | -2.43 | 12.46 | -3.16 | -0.99 | -11.09 | -47.82 | -46.08 | -8.8 | 15.72 |
| L407A×JiR107 | 9.4 | 15.52 | 0.08 | -5.67 | 19.34 | 12.28 | 9.57 | 25.04 | 2.65 |
